# Hybrid Silver(I)-Doped Soybean Oil and Potato Starch Biopolymer Films to Combat Bacterial Biofilms

**DOI:** 10.1021/acsami.2c03010

**Published:** 2022-05-27

**Authors:** Tiago A. Fernandes, Inês F.M. Costa, Paula Jorge, Ana Catarina Sousa, Vânia André, Rafaela G. Cabral, Nuno Cerca, Alexander M. Kirillov

**Affiliations:** † Centro de Química Estrutural, Institute of Molecular Sciences, Departamento de Engenharia Química, Instituto Superior Técnico, Universidade de Lisboa, Av. Rovisco Pais, 1049-001 Lisbon, Portugal; ‡ Centre of Biological Engineering, University of Minho, Campus de Gualtar, 4710-057 Braga, Portugal; § Área Departamental de Engenharia Química, ISEL—Instituto Superior de Engenharia de Lisboa, Instituto Politécnico de Lisboa, R. Conselheiro Emídio Navarro, 1, 1959-007 Lisbon, Portugal

**Keywords:** silver, metal−organic frameworks, biopolymers, antibacterial activity hybrid materials, bacterial biofilms, coordination polymers

## Abstract

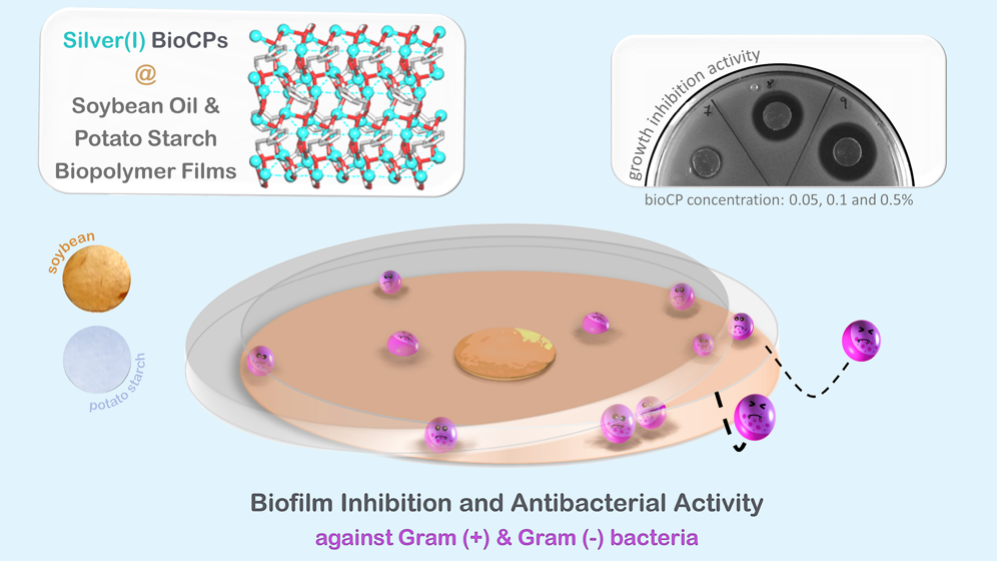

This study describes the preparation, characterization, and antimicrobial properties of novel hybrid biopolymer materials doped with bioactive silver(I) coordination polymers (bioCPs). Two new bioCPs, [Ag_2_(μ_6_-hfa)]_*n*_ (**1**) and [Ag_2_(μ_4_-nda)(H_2_O)_2_]_*n*_ (**2**), were assembled from Ag_2_O and homophthalic (H_2_hfa) or 2,6-naphthalenedicarboxylic (H_2_nda) acids as unexplored building blocks. Their structures feature 2D metal–organic and supramolecular networks with 3,6L64 or sql topology. Both compounds act as active antimicrobial agents for producing bioCP-doped biopolymer films based on epoxidized soybean oil acrylate (SBO) or potato starch (PS) as model biopolymer materials with a different rate of degradability and silver release. BioCPs and their hybrid biopolymer films (**1**@[SBO]_*n*_, **2**@[SBO]_*n*_, **1**@[PS]_*n*_, and **2**@[PS]_*n*_) with a very low loading of coordination polymer (0.05–0.5 wt %) show remarkable antimicrobial activity against *Staphylococcus aureus* and *Staphylococcus epidermidis* (Gram-positive) and *Escherichia coli* and *Pseudomonas aeruginosa* (Gram-negative) bacteria. Biopolymer films also effectively impair the formation of bacterial biofilms, allowing total biofilm inhibition in several cases. By reporting on new bioCPs and biopolymer films obtained from renewable biofeedstocks (soybean oil and PS), this study blends highly important research directions and widens a limited antimicrobial application of bioCPs and derived functional materials. This research thus opens up the perspectives for designing hybrid biopolymer films with outstanding bioactivity against bacterial biofilms.

## Introduction

Bacterial biofilms represent a community of bacteria usually associated with a surface and encased within an extracellular matrix. Biofilms correspond to a very typical form for the growth of bacteria, which are naturally assembled on diverse interface types of materials and environments, including living organisms.^1^ During the formation of biofilms, the adhesion of bacterial cells to the surface occurs with a simultaneous synthesis of a protective matrix.^2,3^ This process enables bacteria to survive under aggressive environments (i.e., heat and treatment with antiseptics and antibiotics) and develop increased resistance, which nowadays constitutes a critical issue in human healthcare.^4−6^ In fact, biofilms are largely responsible for persistent and chronic infection diseases in humans, which are often related with the use of biomaterials (e.g., implants, catheters, and valves).^7,8^ Hence, the prevention of bacterial attachment to surfaces represents one of the most promising strategies to tackle biofilm formation and growth, namely, by investigating alternative functional materials,^9−11^ capable of reducing bacterial adhesion and biofilm formation.

In this regard, bioCPs (bioactive coordination polymers) and bioMOFs (bioactive metal–organic frameworks) attracted a great deal of attention as promising antibacterial agents^12−21^ composed of biocidal metal centers as well as linkers and/or guest species with antimicrobial activity. Among various metals with a potential antibacterial activity, silver is particularly appealing with the most significant bioactivity in addition to a relatively low intrinsic toxicity to human cells.^22−32^

Silver ions, nanoparticles, and coordination compounds are thus well recognized for their bactericidal activity with many partially implicit types of action mechanisms.^27,33−35^ The examples include intrusion of Ag^+^ species into intracellular protein moieties and membranes of bacteria, particularly sulfur-containing proteins and phosphorus-containing deoxyribonucleic acid. Such interactions involving thiol functionalities in essential enzymes contribute to their deactivation, inhibit division of cells, and cause death of cells.^36−38^ Prior results indicate that in bacteria subjected to treatment by Ag^+^ species, DNA can lose the ability to replicate, causing changes in the structures of cell membranes and in the development of minor electron-rich granules incorporating Ag and S elements.^36,38,39^

In pursuit of this discussion and high current perspectives of bioactive coordination polymers and derived materials, the principal aim of this work focused on the synthesis of new silver(I) bioCPs and assessment of their antimicrobial activity after incorporation as antimicrobial dopants into biopolymer films. This research direction tracks a trend for the design of renewable and biodegradable biopolymer materials with diverse applications.^40−43^ As an example, the search for alternative biodegradable packaging products has seen a great development in response to the biological incompatibility of non-biodegradable packaging materials. Biopolymer films that simultaneously comprise an antimicrobial component and derive from the natural bio-based precursors [e.g., soybean oil and potato starch (PS)] may find a high significance in this field.

Specifically, epoxidized soybean oil acrylate (SBO) and PS are considered very promising candidates as organic polymer matrices for the production of antimicrobial biomaterials. Soy protein isolate (SPI) is a promising packaging material derived from the edible oil industry and possesses advantages such as biocompatibility, biodegradability, and film-forming capacity. The limited practical applications of SPI-based films have led to an increased research on epoxide compounds that are able to enhance their mechanical properties. In this regard, SBO represents a particularly interesting substrate for the preparation of biopolymers.^44−46^

Apart from SBO, PS can also act as a low-cost biodegradable polymer matrix to immobilize antimicrobial bioCPs. As a polysaccharide widely used to produce green biomaterials, starch constitutes a renewable and sustainable polymer and represents one of the most commercially available biofeedstocks.^47^ The non-biodegradability of petrochemical-based materials generated a strong demand for “green alternatives”, such as starch-based biodegradable bioplastics. Thus, PS is considered as a highly attractive resource for the production of biopolymer films in the packaging industry.^48−51^

Aiming at fabricating novel functional materials and merging both the synthetic and antimicrobial approaches, the current work reports on the synthesis of new silver(I) bioactive coordination polymers as well as their application as dopants for the preparation of hybrid bioCP-doped biopolymer films based on [SBO]_*n*_ or [PS]_*n*_. Hence, two new bioCPs, [Ag_2_(μ_6_-hfa)]_*n*_ (**1**) and [Ag_2_(μ_4_-nda)(H_2_O)_2_]_*n*_ (**2**), were assembled from homophthalic (H_2_hfa) or 2,6-naphthalenedicarboxylic (H_2_nda) acids as linkers and applied in the production of hybrid biopolymer film materials. These were then screened for their potential to prevent the growth of bacteria and biofilms. By reporting on new bioCPs along with hybrid biopolymer films and their antibacterial and biofilm inhibition properties, this multidisciplinary study not only blends different highly important research directions but also widens a still limited antimicrobial application of coordination polymers and derived biomaterials.

## Results and Discussion

### Preparation of bioCPs

New silver(I) bioCPs formulated as [Ag_2_(μ_6_-hfa)]_*n*_ (**1**) and [Ag_2_(μ_4_-nda)(H_2_O)_2_]_*n*_ (**2**) were self-assembled from an acetonitrile–methanol reaction mixture composed of silver(I) oxide, homophthalic (H_2_hfa) or 2,6-naphthalenedicarboxylic (H_2_nda) acid, 2-dimethylaminoethanol (template), and aqueous ammonium hydroxide (Figure S1, Supporting Information). Both products **1** and **2** were isolated as air-stable crystalline compounds having a mean size of particles of 66 and 51 μm, respectively (Figures S2 and S6). The formulation and structures of the obtained compounds were confirmed by C/H/N analyses, IR (Figures S7 and S8) and NMR spectroscopies, and X-ray diffraction methods (SCXD and PXRD, Figures S20 and S21).

### Preparation of Hybrid BioCP-Doped Biopolymer Films

**Figure 1:**
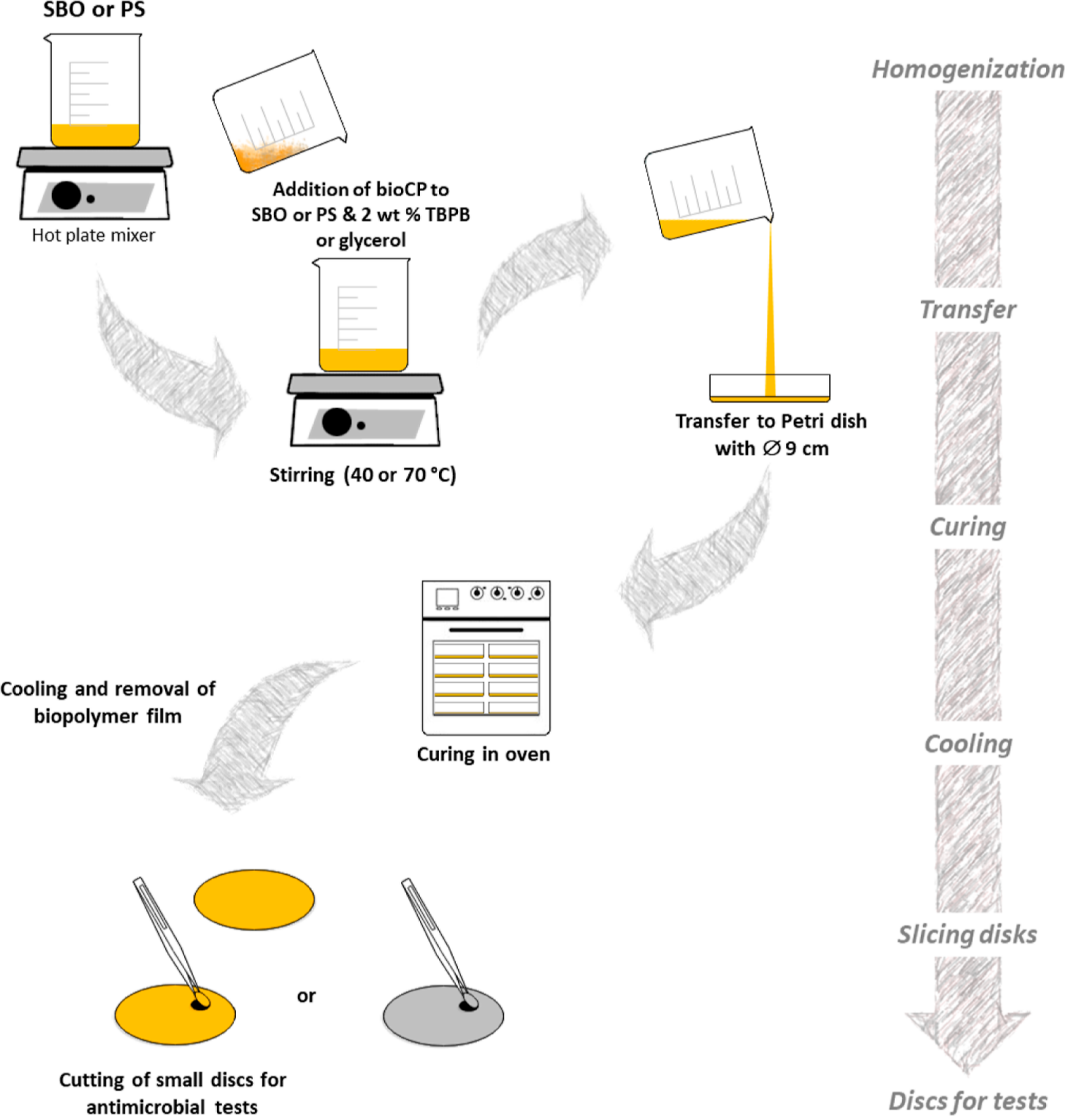
Preparation of bioCP-doped [SBO]_*n*_ and [PS]_*n*_ biopolymer films.

**Figure 2:**
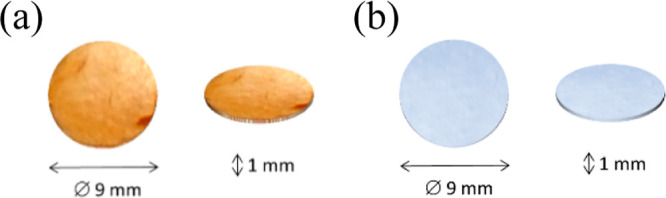
Sample coupons for (a) [SBO]_*n*_ and (b) [PS]_*n*_ biopolymer films.

Two types of hybrid biopolymer films were fabricated by dispersing very low amounts of bioCPs in the biopolymer precursors based on the epoxidized SBO, or PS with glycerol, followed by polymerization in Petri dishes (Figures 1 and S4). The examples of sample coupons for [SBO]_*n*_ and [PS]_*n*_ biopolymer films that were cut and used for antimicrobial studies are shown in Figure 2. Low loadings (0.05, 0.01, and 0.5 wt %) of bioCPs were explored, giving origin to the biopolymer thin films (∼1 mm thickness) abbreviated as **1**-0.5%@[SBO]_*n*_, **1**-0.1%@[SBO]_*n*_, and **1**-0.05%@[SBO]_*n*_; **2**-0.5%@[SBO]_*n*_, **2**-0.1%@[SBO]_*n*_, and **2**-0.05%@[SBO]_*n*_ (SBO series); as well as **1**-0.5%@[PS]_*n*_ and **2**-0.5%@[PS]_*n*_ (PS series) (Figure S5). For comparison, the negative control ([SBO]_*n*_ and [PS]_*n*_) as well as the positive control (Ag_2_O-0.05%@[SBO]_*n*_, Ag_2_O-0.1%@[SBO]_*n*_, Ag_2_O-0.5%@[SBO]_*n*_, and Ag_2_O-0.5%@[PS]_*n*_) biopolymer films were also fabricated. The obtained materials were characterized by ATR-FTIR (Figures S9–S14) and SEM–EDX (Figures 5, S15, and S16). The latter revealed a generally uniform distribution of bioCPs, although these may occasionally be concentrated in areas containing larger crystalline particles. Water absorption and stability of films in PBS medium were also evaluated in addition to silver ion release experiments (Table S1 and Figures S17 and S18). In fact, both types of biopolymer films show a gradual disaggregation (limited stability) along time in PBS medium (Figure S17, Supporting Information) and feature a minor release of Ag^+^ ions after 24 h, namely, 10–20 and 49–57 μg/L, for [SBO]_*n*_ and [PS]_*n*_ films doped by 0.5% bioCPs, respectively. We would like to emphasize that the materials should not be intact to show antimicrobial performance.

Similar to a majority of related CPs or MOFs, both the obtained bioCPs are marginally soluble in H_2_O and in reaction medium during the film formation. This makes more difficult the homogeneous distribution of coordination polymers within the biopolymer films. However, in contrast to discrete soluble complexes or silver salts, the release of silver ions from bioCP-based films is significantly slower, which represents an advantage of these materials in terms of relative stability and possible long-term use. Both carboxylic acid ligands in **1** and **2** were chosen given their unexplored use as building blocks for assembling bioCPs and due to their different hydrolipophilic properties, for example, log *P* = 1.18 for homophthalic acid and log *P* = 2.80 for 2,6-naphthalenedicarboxylic acid. However, when these ligands are coordinated to silver, they form almost insoluble bioCPs, with a different rate of silver ion release.

### Structural Description of bioCPs **1** and **2**

**Figure 3:**
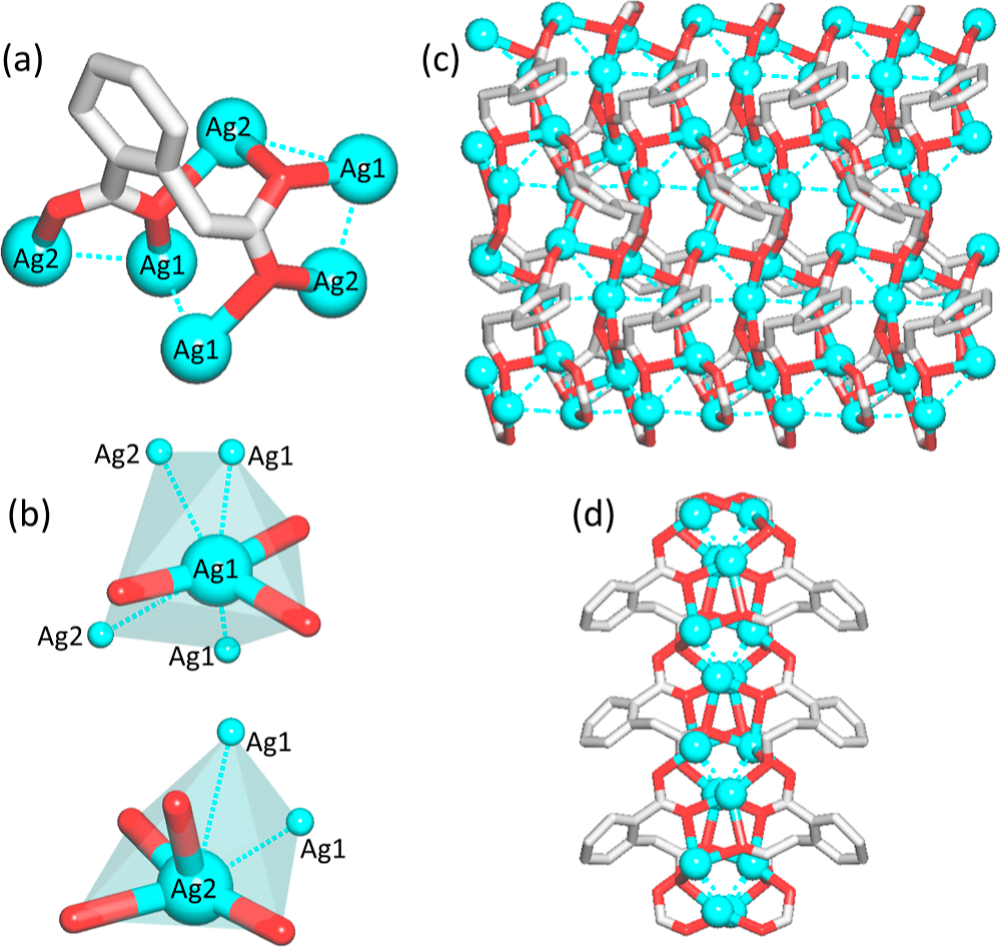
Structural fragments of [Ag_2_(μ_6_-hfa)]_*n*_ (**1**). (a) Coordination mode of μ_6_-hfa^2–^ ligand. (b) Coordination environment of silver atoms including argentophilic interactions (dotted cyan lines). (c,d) Front (c) and side (d) view of 2D double layer. Further details: (a–d) Ag (cyan), C (gray), and O (red); views along the *c* (c) and *a* (d) axes.

The structure of [Ag_2_(μ_6_-hfa)]_*n*_ (**1**) discloses an intricate 2D metal–organic double layer (Figure 3), which is constructed from two structurally distinct Ag1/Ag2 centers and a μ_6_-homophthalate^2–^ linker (Figure 3a,b). This linker simultaneously binds to three Ag1 and three Ag2 atoms by carboxylate groups that act in the μ_3_-bridging tridentate and μ_4_-bridging tetradentate modes. The Ag1 center is 3-coordinate featuring a distorted trigonal planar arrangement of three carboxylate O donor atoms with the Ag1–O distances in the 2.1677(1)–2.4711(1) Å range. The Ag2 center is 4-coordinate by four oxygen atoms coming from three μ_6_-hfa^2–^ blocks with the Ag2–O bonds varying in the 2.2193(1)–2.6405(1) Å interval. In addition, both the Ag1 and Ag2 centers participate in several weak argentophilic interactions, wherein the Ag···Ag distances are in the 2.8204(6)–3.1861(5) Å interval (Figure 3b). These interactions reinforce the 2D metal–organic network (Figure 3c,d) that is driven by the μ_6_-hfa^2–^ linkers and features a 3,6L64 topology (Figure S3a,b). Despite being rather simple and commercially available, homophthalic acid has not yet been applied for designing silver complexes and CPs as evidenced by CSD search. Hence, bioCP **1** represents a unique example of Ag(I) coordination polymer derived from H_2_hfa.

**Figure 4:**
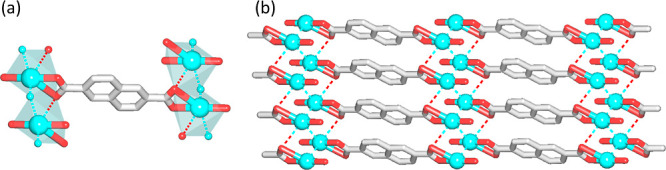
Structural fragments of [Ag_2_(μ_4_-nda)(H_2_O)_2_]_*n*_ (**2**). (a) Coordination mode of μ_4_-nda^2–^ ligand and environment of Ag1 centers; weaker argentophilic Ag···Ag and Ag···O interactions are shown as dotted lines. (b) 2D supramolecular layer. Further details: Ag (cyan), C (gray), and O (red); (b) view along the *c* axis.

The structure of [Ag_2_(μ_4_-nda)(H_2_O)_2_]_*n*_ (**2**) is assembled from the symmetry equivalent Ag1 atoms, μ_4_-nda^2–^ spacers, and terminal water ligands (Figure 4). Note that the discussion is based on the major occupancy Ag atom (Ag1), but similar conclusions can be drawn for the Ag2 center. The Ag1 atoms are surrounded by three oxygen atoms, two coming from the μ_4_-nda^2–^ blocks and one from the H_2_O ligand with the Ag1–O bonds ranging from 2.1203(3)(3) to 2.7146(4) Å (Figure 4a,b). There are also weaker Ag···Ag [3.01–3.25 Å] and Ag···O [3.26 Å] interactions which, along with the μ_4_-nda^2–^ linkers, result in the assembly of a two-dimensional supramolecular net (Figure 4b) with an sql topology (Figure S3c). The compound **2** widens the types of CPs constructed from the nda^2–^ linkers.^52,53^

### Morphological Characterization of bioCP-Doped Biopolymer Films

**Figure 5:**
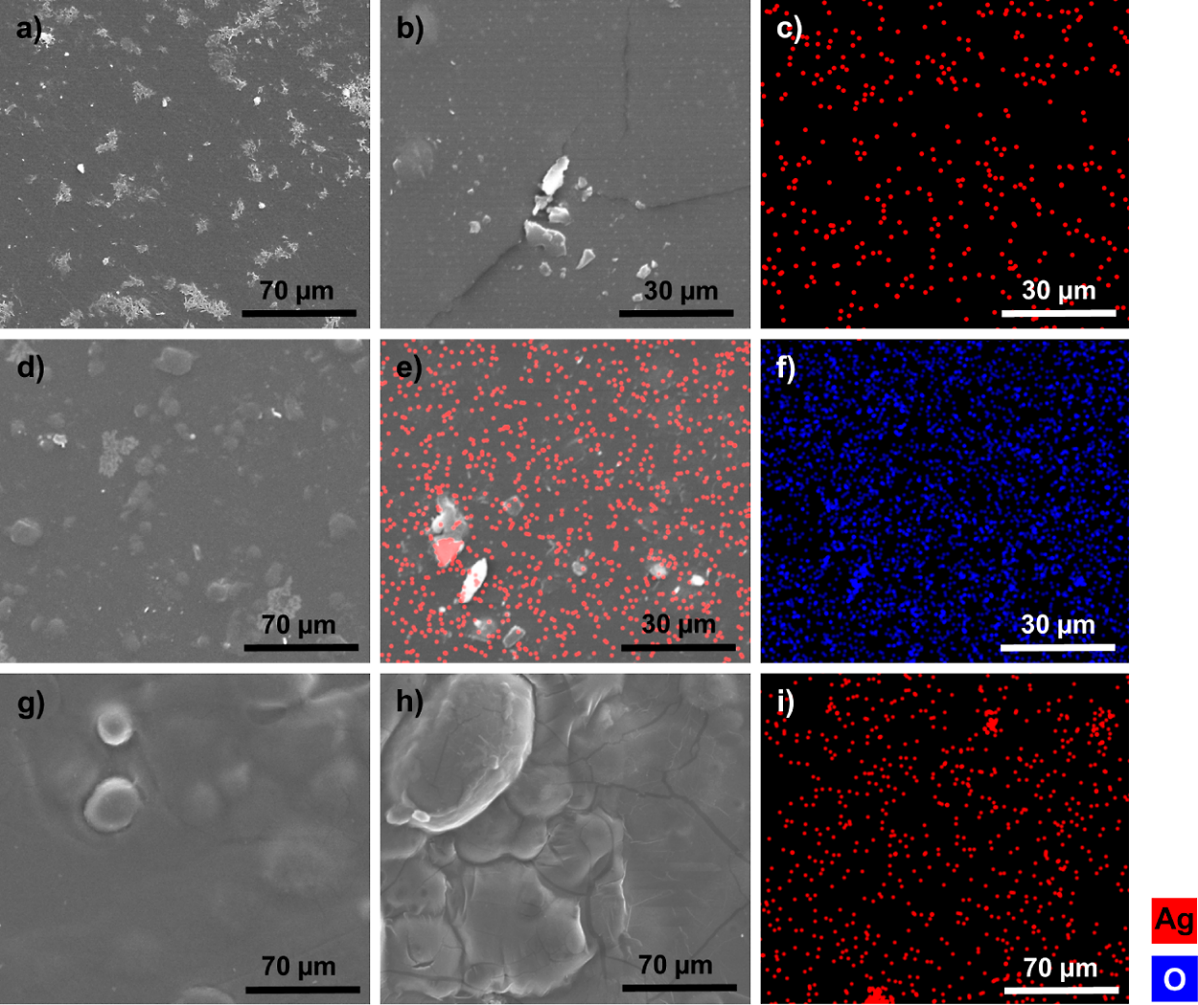
Morphological characterization of [SBO]_*n*_ and [PS]_*n*_ films by SEM–EDX. SEM images: (a) SBO film; (b) **1**-0.5%@[SBO]_*n*_; (c) **1**-0.5%@[SBO]_*n*_ [the same region as (b)] with EDX analysis of Ag distribution; (d) **2**-0.5%@[SBO]_*n*_, where bioCP particles can be seen throughout the material; (e) **2**-0.5%@[SBO]_*n*_ with EDX analysis of Ag distribution; (f) **2**-0.5%@[SBO]_*n*_ (the same region as e) with EDX analysis of O distribution; (g) [PS]_*n*_ film; (h) **1**-0.5%@[PS]_*n*_, where bioCP particles can be seen throughout the material; (i) **1**-0.5%@[PS]_*n*_ (the same region as h) with EDX analysis of Ag distribution. Images (a), (d), (i), and (h) were obtained at 1000× magnification and (b,c,e,f,i) at 500× magnification.

The bioCP-doped [SBO]_*n*_ and [PS]_*n*_ biopolymer films were examined by SEM–EDX (Figure 5) to further evaluate their morphology and incorporation of bioCPs **1** or **2** into the films. As observed in Figure 5b,e, coordination polymers **1** and **2** were directly embedded within the [SBO]_*n*_ matrix, also being present at the surface of the films (Figure 5b). The material was then evaluated by SEM–EDX using a silver analysis probe for determining Ag distribution (Figure 5c,e). From the Ag overlay on the SEM image of the **2**-0.5%@[SBO]_*n*_ film (Figure 5e), it can be seen that silver is well distributed and only occasionally concentrated in areas containing larger crystalline particles of bioCP **2**. The oxygen distribution does not provide additional details because O element is present in both the polymer matrix and bioCPs (Figure 5f). Figure 5g shows a morphological characterization of the [PS]_*n*_ film. Figure 5h,i shows the same region of **1**-0.5%@[PS]_*n*_, where bioCP particles can be seen throughout the material, which is evidenced by EDX analysis of Ag distribution. In general, the films are quite uniform (Figure 5c,e,i), although it is possible to occasionally observe areas containing increased bioCP concentrations (Figures S15 and S16, Supporting Information). However, taking into account the sample coupon as a whole, any eventual deviation in the distribution of bioCPs is irrelevant, as confirmed by the reproducibility of biological assays that were made in triplicate.

### Thermogravimetric Analysis

The thermal stability of bioCP-doped and undoped [SBO]_*n*_ and [PS]_*n*_ biopolymer films was investigated by TGA under nitrogen flow (Figure S19, Supporting Information). All [SBO]_*n*_-based samples display similar patterns with one major degradation stage between 300 and 470 °C, corresponding to a 90% weight loss. For [PS]_*n*_-based samples, there are several thermal effects with major mass losses in the 50–250 and 250–350 °C intervals, eventually corresponding to a release of absorbed H_2_O/glycerol and the decomposition of biopolymers, respectively.^54,55^ The third step of weight loss (350–700 °C) can be assigned to final carbonization. Other difference in the TGA patterns observed between the bioCP-doped samples and sole [PS]_*n*_ might be regarded to a different content of absorbed water and glycerol present in these samples.

### Antibacterial Activity

**Figure 6:**
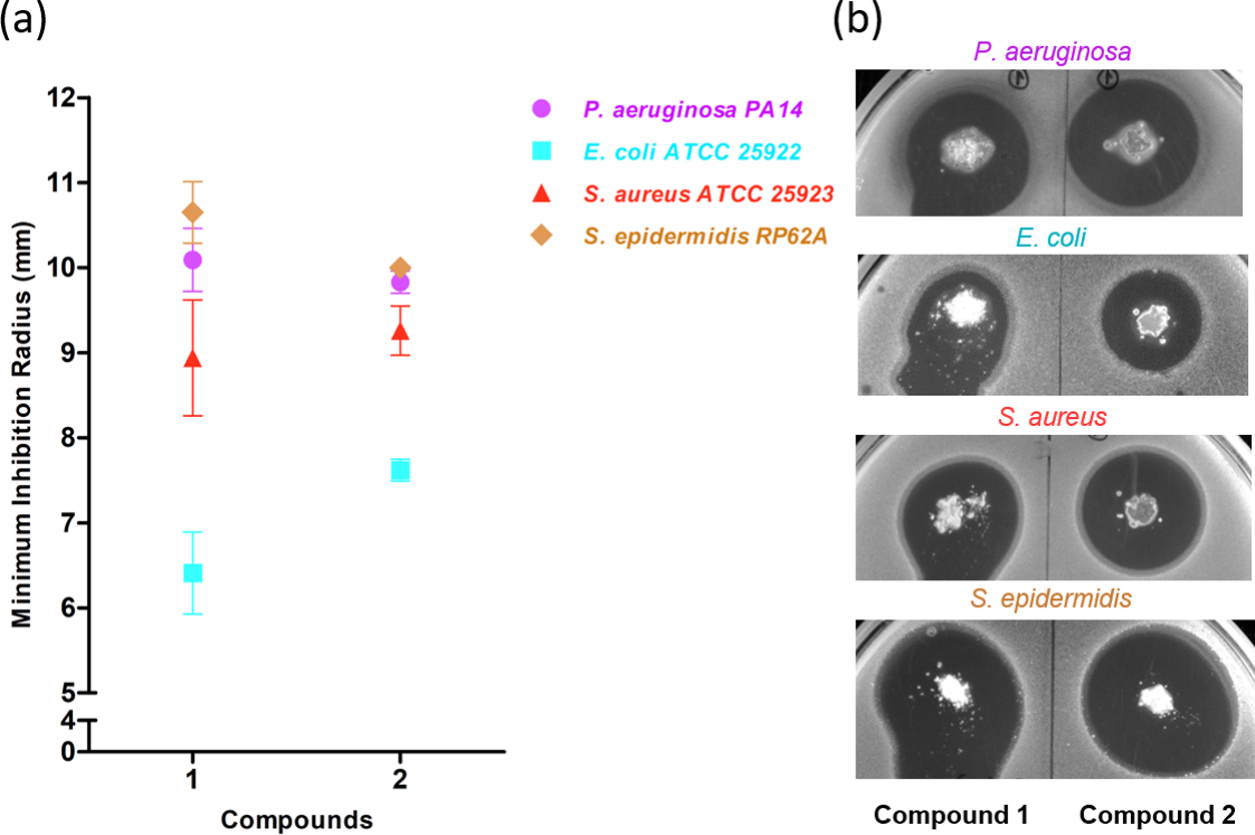
(a) Minimum inhibition radius (mean ± SD) showing the normalized activity of **1** and **2** against *P. aeruginosa* and *E. coli* (Gram-negative) and *S. aureus* and *S. epidermidis* (Gram-positive) bacteria. (b) Examples of the obtained halos representing bacterial growth inhibition.

The obtained bioCPs exhibited antibacterial behavior against all tested species (Figure 6). In terms of susceptibility, the following rough trend was observed: *Escherichia coli* < *Staphylococcus aureus* < *Pseudomonas aeruginosa* < *Staphylococcus epidermidis*. No link of antibacterial efficiency with Gram classification was observed. Compound **1** had higher antibacterial action against *P. aeruginosa* and *S. epidermidis* in comparison with **2**, which can be evidenced by larger radii of inhibition. For *S. aureus*, the antibacterial activities of **1** and **2** were comparable (Figure 6). In turn, compound **2** was more effective against *E. coli*.

**Figure 7:**
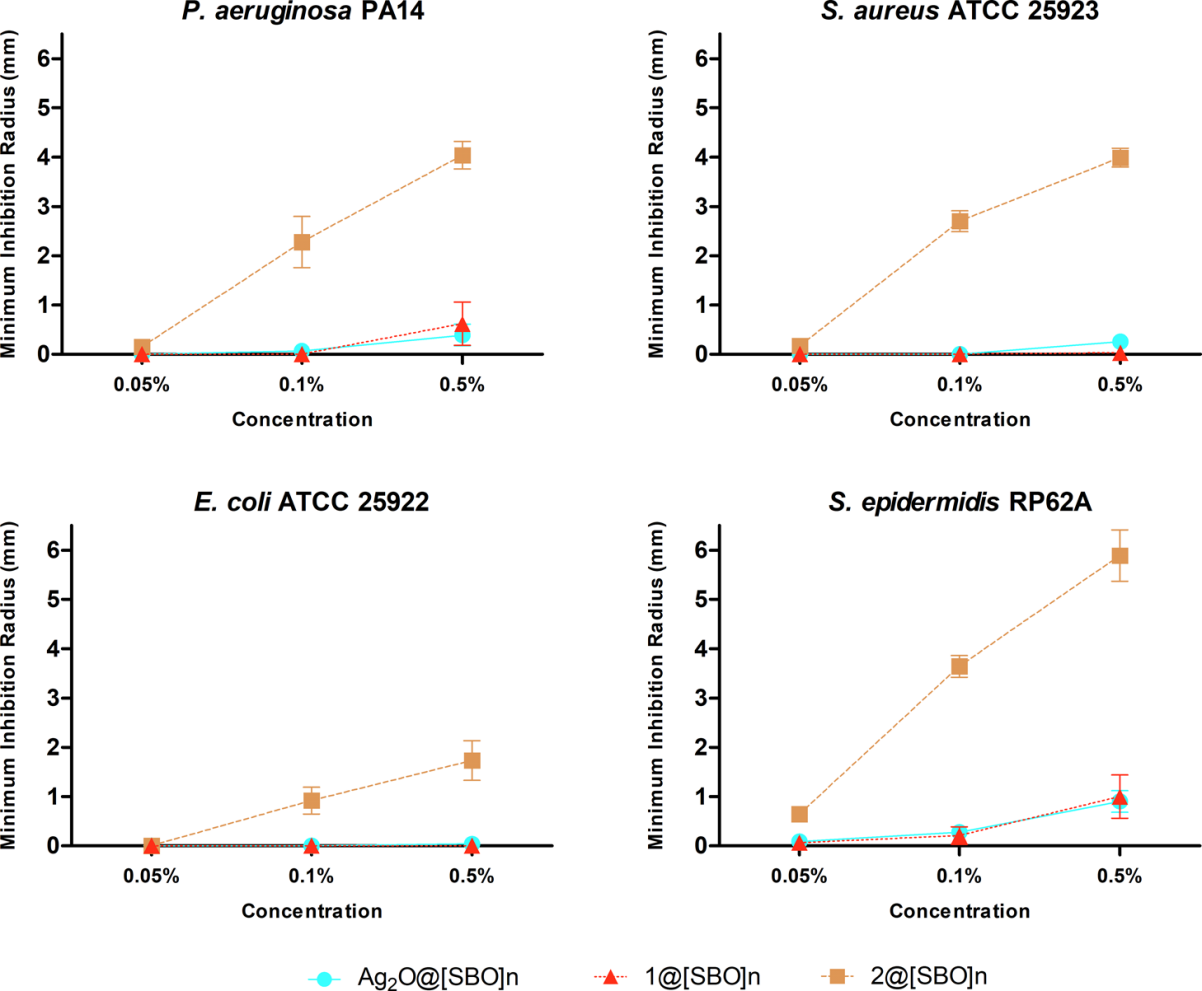
Normalized activity of [SBO]_*n*_ biopolymer films containing varying concentrations of Ag_2_O (Ag_2_O@[SBO]_*n*_) and compounds **1** (**1**@[SBO]_*n*_) and **2** (**2**@[SBO]_*n*_) against *P. aeruginosa* and *E. coli* (Gram-negative) and *S. aureus* and *S. epidermidis* (Gram-positive) bacteria.

Concerning the antibacterial activity of the Ag-doped [SBO]_*n*_ films, **2**@[SBO]_*n*_ stood out as the most effective against all four bacterial strains, with a concentration-dependent efficacy (Figure 7). This is different from the above-discussed data for free compounds, wherein bioCP **1** revealed a superior efficiency (except for *E. coli*). This difference might be regarded to distinct diffusion patterns of silver compounds in the [SBO]_*n*_ film. In fact, a higher Ag^+^ release rate of 20 μg·L^–1^ was observed for **2**@[SBO]_*n*_ versus **1**@[SBO]_*n*_ after 24 h in PBS (Figure S18).

Both **1**@[SBO]_*n*_ and Ag_2_O@[SBO]_*n*_ had little activity in comparison with **2**@[SBO]_*n*_. All the three films had similar ineffectiveness at 0.05 wt % loading. The least susceptible bacteria to **2**@[SBO]_*n*_ was *E. coli*, while *S. epidermidis* was the most susceptible.

**Figure 8:**
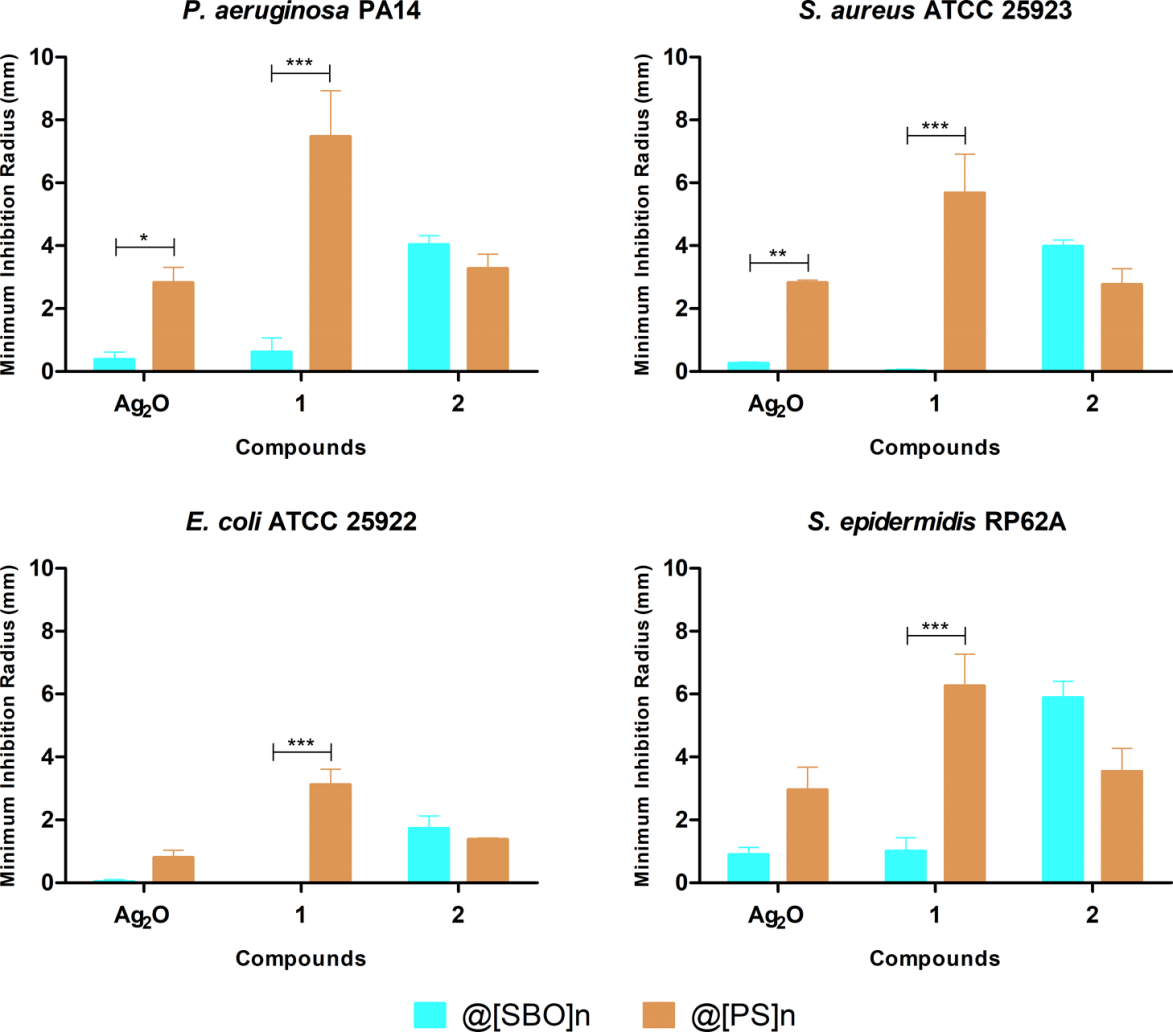
Normalized activity of [SBO]_*n*_*vs* [PS]_*n*_ biopolymer films containing 0.5% of Ag_2_O and compounds **1** and **2** against *P. aeruginosa* and *E. coli* (Gram-negative) and *S. aureus* and *S. epidermidis* (Gram-positive) bacteria. Significant statistical differences were found using the two-way ANOVA and subsequent Bonferroni’s multiple comparisons test with 95% confidence interval, represented as *P ≤ 0.05; **P ≤ 0.01; and ***P ≤ 0.001.

To compare an antimicrobial efficiency of silver(I) compounds in [SBO]_*n*_ versus [PS]_*n*_ films, an antibacterial assay was performed for both biopolymers encompassing the most effective concentration of bioCPs (0.5%). The antimicrobial activity of compound **1** was substantially higher when loaded into [PS]_*n*_ rather than [SBO]_*n*_ films. Similarly, Ag_2_O also showed some increased activity in [PS]_*n*_ films. In turn, compound **2** had a resembling antimicrobial activity in both films (Figure 8). The differences encountered between the two films are related with their composition, namely, a higher water content in [PS]_*n*_ versus residual water in [SBO]_*n*_, as observed by FTIR (Figures S9 and S12), which may lead to a quicker solubilization and/or migration of bioCPs from [PS]_*n*_. In fact, this was further confirmed by a faster Ag^+^ release to the media from both **1**-0.5%@[PS]_*n*_ and **2**-0.5%@[PS]_*n*_ samples if compared with Ag-doped [SBO]_*n*_ samples (Figure S18).

The increase in mass as a percentage of dry mass for both types of biopolymer films was studied to determine water absorption. All samples were kept in water solution for 24 h. Based on the results (Table S1), it is clear that water absorption of 60% is higher for [PS]_*n*_ films in comparison to a residual 1% absorption of [SBO]_*n*_ films. As expected, higher water absorption well correlates with an affinity of [PS]_*n*_ films to water as discussed above. All these findings well rationalize an observed bioactivity of different biopolymer films.

The amount of silver ions released, from both materials (with 0.5% concentration of bioCP, **1**-0.5%@[SBO]_*n*_, and **1**-0.5%@[PS]_*n*_), to PBS solution was determined by ICP-OES. In addition to proving a stability of materials, it revealed that only 0.06% of all silver present in the sample was released after 24 h from **1**@[SBO]_*n*_, in contrast to 0.36% for **1**@[PS]_*n*_, which corresponds to 10 and 57 μg·L^–1^, respectively. Similar results were obtained for **2**@[SBO]_*n*_ and **2**@[PS]_*n*_, showing that [PS]_*n*_ films release 6 times more silver ions to the aqueous medium within 24 h (Figures S17 and S18). The different Ag^+^ release rates observed for bioCP-doped [SBO]_*n*_ and [PS]_*n*_ biopolymer films are associated with the intrinsic properties (permeability, stability, degradability, and water absorption) of the biopolymer films. An effect of eventual interaction of compounds **1** and **2** with [SBO]_*n*_ and [PS]_*n*_ on release rate of Ag^+^ is negligible, given also a very low content (only 0.05–0.5%) of these bioCPs used as dopants.

A number of Ag(I) coordination polymers or metal–organic frameworks with various Ag–O, Ag–N, and/or Ag–P coordination environment types have been documented as antimicrobial compounds, with significant activity against the types of bacteria tested in the present study (Table S2, Supporting Information).^18,19,21,56−58^ Given the difference in the content of antimicrobial components and the use of distinct assays for evaluation of bioactivity, a quantitative comparison of the observed activities is not feasible. Besides, the normalized antimicrobial activity of biopolymer films doped with bioCPs is generally comparable or even superior than that shown by the reference films doped with Ag_2_O and AgNO_3_ (Table S3, Supporting Information). Especially, the **2**-0.1%@[SBO]_*n*_ films exhibit a higher activity for all the tested types of bacteria if compared to the respective AgNO_3_-0.1%@[SBO]_*n*_ films. Hence, important advantages of the bioCPs and materials reported in the present work concern (1) a usage of inexpensive soybean oil and PS as precursors for biopolymers, (2) a pronounced antimicrobial activity of the obtained films despite a very low loading of bioCPs (0.05–0.5%), (3) a simple generation of compounds **1** and **2** from commercially available reagents, and (4) a possibility of applying the biopolymer films to inhibit the growth of bacterial biofilms.

### Biofilm Inhibition Activity

**Figure 9:**
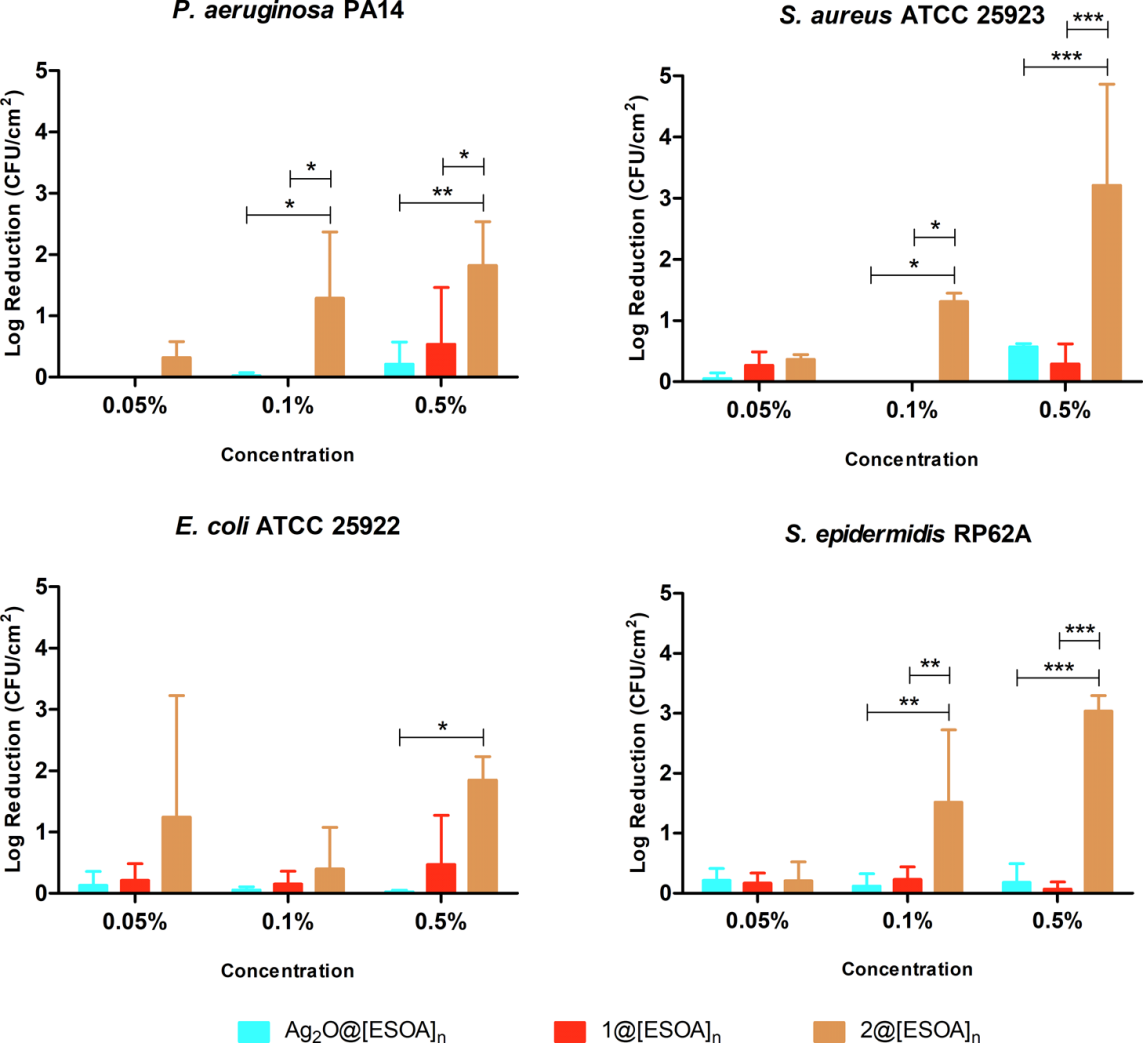
Normalized biofilm inhibition activity of [SBO]_*n*_ biopolymer films containing varying concentration of Ag_2_O (Ag_2_O@[SBO]_*n*_) and compounds **1** (**1**@[SBO]_*n*_) and **2** (**2**@[SBO]_*n*_), against *P. aeruginosa* and *E. coli* (Gram-negative) and *S. aureus* and *S. epidermidis* (Gram-positive) bacteria. Significant statistical differences were found using the two-way ANOVA and subsequent Bonferroni’s multiple comparisons test with 95% confidence interval, represented as *P ≤ 0.05; **P ≤ 0.01; and ***P ≤ 0.001.

The Ag-doped [SBO]_*n*_ films were also tested for their ability to prevent biofilm formation. Similar to its antibacterial activity, **2**@[SBO]_*n*_ was overall the most effective in partially preventing all the four bacterial species from forming biofilms on its surface (Figure 9). The Gram-positive bacteria, *S. epidermidis* and *S. aureus*, seem to be the most susceptible to **2**@[SBO]_*n*_, with reductions above 3 log (99.9% bacterial growth reduction). The effect of **1**@[SBO]_*n*_ was much lower, with some effect (although lower than 1 log reduction, i.e., <90%) at the highest concentration against *P. aeruginosa* and *E. coli* (Gram-negative) bacteria, while the activity of Ag_2_O@[SBO]_*n*_ as a positive control was virtually non-existent.

**Figure 10:**
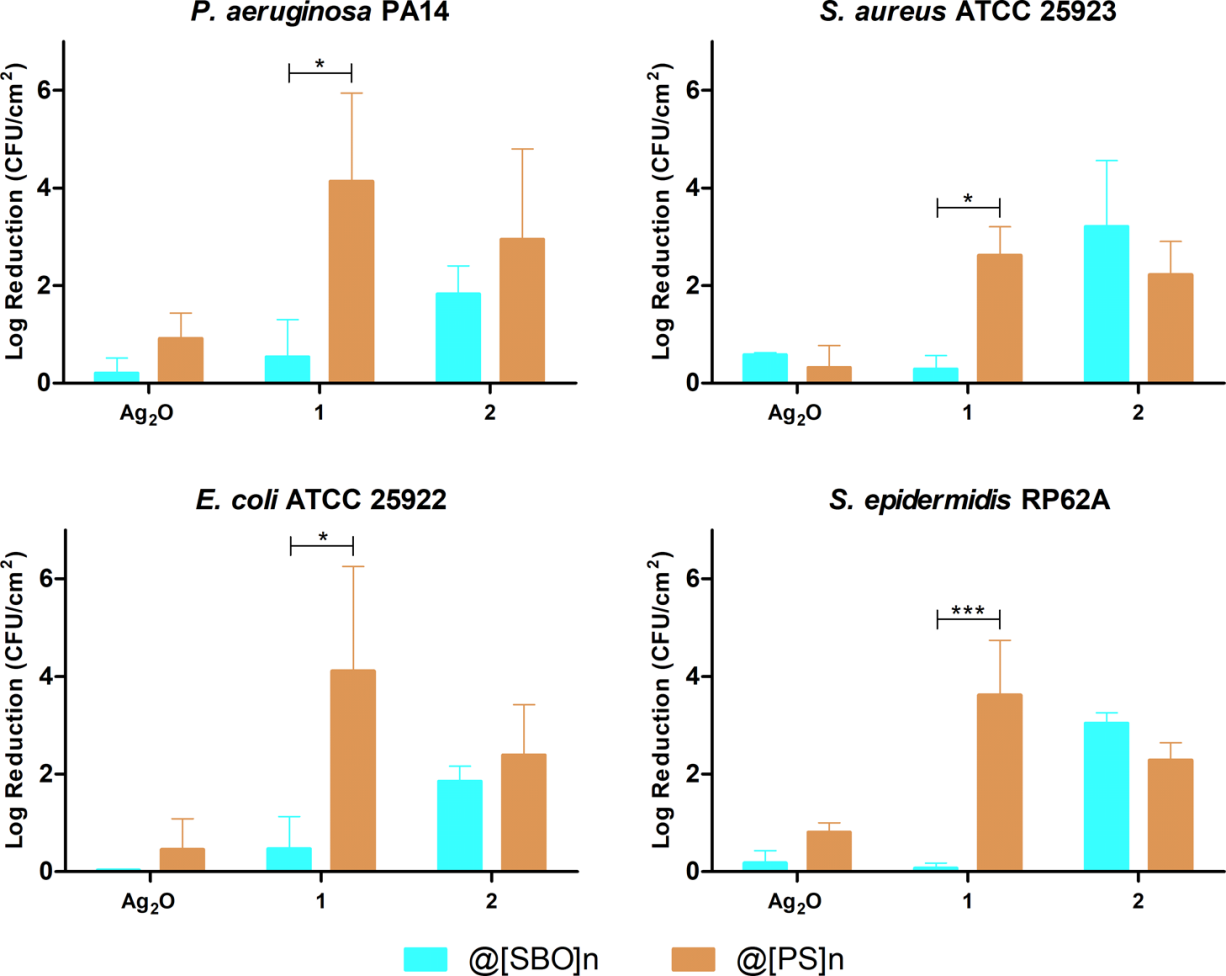
Normalized biofilm inhibition activity of [SBO]_*n*_*vs* [PS]_*n*_ biopolymer films doped with 0.5% of Ag_2_O and compounds **1** and **2** against *P. aeruginosa* and *E. coli* (Gram-negative) and *S. aureus* and *S. epidermidis* (Gram-positive) bacteria. Significant statistical differences were found using the two-way ANOVA and subsequent Bonferroni’s multiple comparisons test with 95% confidence interval, represented as *P ≤ 0.05; **P ≤ 0.01; and ***P ≤ 0.001.

In parallel with what was observed for the antibacterial activity, the efficacy of the bioCP-doped [SBO]_*n*_ films in preventing biofilm formation was compared with that of the respective [PS]_*n*_ films. Overall, the efficacy of Ag_2_O and compound **2** was similar for both films, while the efficacy of compound **1** was much higher in [PS]_*n*_ films (Figure 10). This correlates with what was observed for the antibacterial activity (Figure 8) and silver release tests (Figure S18).

## Experimental Section

### Self-Assembly Synthesis and Characterization of **1** and **2**

#### [Ag_2_(μ_6_-hfa)]_*n*_ (**1**)

Acetonitrile (7 mL) and methanol (3 mL) were added to Ag_2_O (0.232 g, 1 mmol). Then, homophthalic acid (H_2_hfa, 0.180 g, 1 mmol), 2-dimethylaminoethanol (Hdmea, 1.0 mmol, 0.1 mL), and 8.0 mL of aq NH_4_OH (33%) were added, and the reaction mixture was stirred for 30 min at room temperature, followed by filtration (filter paper). An obtained filtrate was slowly evaporated in a vial in air. Colorless microcrystals (including suitable for X-ray diffraction) appeared in 1–2 weeks, which were separated and air-dried to afford bioCP **1** in 59% yield relatively to silver(I) oxide. BioCP **1** is barely soluble in water (0.065 mg·mL^–1^). Analysis calculated (%) for **1**, C_9_H_6_Ag_2_O_4_ + 0.5CH_3_OH + 0.25CH_3_CN (**1** + 0.5CH_3_OH + 0.25CH_3_CN): C, 28.59; H, 2.10; N, 0.83. Found: C, 29.04; H, 1.63; N, 0.47. FTIR-ATR (cm^–1^): 3207 m ν(H_2_O), 3065 m ν(H_2_O), 1606 m, 1583 m, 1539 m ν_as_(COO), 1423 s, 1309 s ν_s_(COO), 1145 w, 1041 w, 920 w, 827 w, 747 m, 716 s, 668 s cm^–1^. ^1^H NMR (DMSO-*d*_6_, 400 MHz): δ ppm 7.59 (1H, d, *J* = 7.1 Hz), 7.27 (1H, t, *J*_AB_ = 7.2, *J*_BA_ = 7.4 Hz), 7.21 (2H, t, *J*_AB_ = 7.4, *J*_BA_ = 7.8 Hz), 7.14 (1H, d, *J* = 7.5 Hz), 3.52 (2H, s, CH_2_). The crystal structure of **1** was determined by X-ray diffraction (Table 1); for details, see the Supporting Information. CCDC 2055525.

**Table 1:** Crystal Data and Structure Refinement Details for **1** and **2**

	**1**	**2**
formula	C_9_H_6_Ag_2_O_4_	C_6_H_5_AgO_3_
fw	393.88	232.97
crystal form, color	block, colorless	needle, colorless
crystal size (mm)	0.3 × 0.04 × 0.02	0.2 × 0.06 × 0.04
crystal system	orthorhombic	monoclinic
space group	*Pbca*	C2/c
*a*, Å	6.2548(2)	27.973(3)
*b*, Å	10.4480(3)	3.8045(5)
*c*, Å	28.3300(8)	13.0369(16)
α, deg	90	90
β, deg	90	107.283(4)
γ, deg	90	90
*Z*	8	8
*V*, Å^3^	1851.37(10)	1324.8(3)
*T*, K	293	293
*D*_c_, g cm^–3^	2.826	2.336
μ(Mo Kα), mm^–1^	4.216	2.977
θ range (deg)	3.261–32.574	3.051–26.352
refl. collected	26,148	15,269
independent refl.	3340	1352
*R*_int_	0.0342	0.0275
*R*_1_^a^, wR_2_^b^ [*I* ≥ 2σ(*I*)]	0.0387, 0.0724	0.0211, 0.0605
GOF on *F*^2^	1.184	1.095

a *R*_1_ = Σ||*F*_o_| – |*F*_c_||/Σ|*F*_o_|.

b w*R*_2_ = [Σ[w(*F*_o_^2^ – *F*_c_^2^)^2^]/Σ[w(*F*_o_^2^)^2^]]^1/2^.

#### [Ag_2_(μ_4_-nda)(H_2_O)_2_]_*n*_ (**2**)

Compound **2** was synthesized by a method similar to **1** but utilizing 2,6-naphthalenedicarboxylic acid (H_2_nda, 0.216 g, 1 mmol) in place of H_2_hfa. Pale yellow microcrystals (including suitable for X-ray diffraction) appeared in 1–2 weeks, which were separated and air-dried to afford bioCP **2** in 47% yield relatively to silver(I) oxide. BioCP **1** is barely soluble in water (0.089 mg·mL^–1^). Analysis calculated (%) for **2**, C_12_H_6_O_4_Ag_2_(H_2_O)_2_ + CH_3_CN (**2** + CH_3_CN): C, 33.17; H, 2.58; N, 2.76. Found: C, 33.44; H, 1.95; N, 2.86. FTIR-ATR (cm^–1^): 3322 m ν(H_2_O), 3209 m ν(H_2_O), 1602 w, 1539 s ν_as_(COO), 1490 m, 1383 s, 1348 s ν_s_(COO), 1199 m, 1138 w, 1098 w, 910 w, 831 w, 777 s cm^–1^. ^1^H NMR (400 MHz, DMSO-*d*_6_): δ ppm 8.45 (2H, s), 8.00 (2H, d, *J* = 8.3 Hz), 7.93 (2H, d, *J* = 7.4 Hz). The crystal structure of **2** was determined by X-ray diffraction (Table 1); for details, see the Supporting Information. CCDC 2055526.

### Synthesis of bioCP-Doped [SBO]_*n*_ Films

Prior to incorporation into the [SBO]_*n*_, coordination polymers **1** or **2** were ground into fine-powder solids. Then, bioCPs were introduced in different quantities to SBO (6.5 g) at loadings of 0.05–0.5 wt %, namely, SBO/bioCP 1000:0.5, 1000:1, 1000:5, and 1000:0 (control SBO sample). The mixtures obtained were stirred for 5 min at 40 °C for uniform dispersion of bioCPs in SBO; then, *tert*-butyl peroxybenzoate (TBPB, 2 wt % relatively to SBO) was added. After additional homogenization during 2 min, the mixtures were placed to 9 cm Petri dishes for the preparation of thin films of biopolymers. Polymerization of SBO was carried out by heating Petri dish samples in an oven for 2 h at 120 °C, in addition to subsequent treatment at 160 °C for 4 h. After cooling, the biopolymers were detached from Petri dishes to produce thin bioCP-doped biopolymer films that were abbreviated as follows: **1**-0.5%@[SBO]_*n*_, **1**-0.1%@[SBO]_*n*_, and **1**-0.05%@[SBO]_*n*_ and **2**-0.5%@[SBO]_*n*_, **2**-0.1%@[SBO]_*n*_, **2**-0.05%@[SBO]_*n*_, and [SBO]_*n*_ (control sample). For comparative purposes, the related types of the Ag_2_O-doped (i.e., Ag_2_O-0.05%@[SBO]_*n*_, Ag_2_O-0.1%@[SBO]_*n*_, and Ag_2_O-0.5%@[SBO]_*n*_) and AgNO_3_-doped biopolymer films were also prepared following the above-described procedure. FTIR-ATR (cm^–1^): 2019 vs, 2850 s, 1730 vs, 1175 vs, 1097 s, 1051 s, 809 w, 722 m cm^–1^.

### Preparation of bioCP-Doped [PS]_*n*_ Films

Prior to incorporation into the [PS]_*n*_-based biopolymers, bioCPs **1** and **2** were ground into fine-powder solids. A mixture of PS (1 g), distilled water (10 mL), and glycerol (0.8 mL) (1:10:0.8 mass ratio) was prepared by mixing PS with water for 3 min at 80 °C, followed by adding glycerol. Then, compounds **1** or **2** were added, and the mixtures obtained were stirred for 15 min at 70 °C until reaching a complete homogenization. Then, the mixtures were poured into Petri dishes (0.09 m of diameter) and kept at 50 °C in an oven for polymerization for 24 h. The malleable biopolymer films were detached from Petri dishes to furnish the bioCP-doped [PS]_*n*_ biopolymer films **1**-0.5%@[PS]_*n*_ and **2**-0.5%@[PS]_*n*_. In parallel experiments, the control [PS]_*n*_ and Ag_2_O-0.5%@[PS]_*n*_ films were prepared for comparative purposes. FTIR-ATR (cm^–1^): 3300 br, 2936 m, 2886 m, 1645 w, 1456 w, 1417 w, 1335 w, 1151 m, 1105, m, 1078 m, 1020 vs, 999 vs, 923 m, 852 m cm^–1^.

## Conclusions

Two new silver(I) bioCPs, [Ag_2_(μ_6_-hfa)]_*n*_ (**1**) and [Ag_2_(μ_4_-nda)(H_2_O)_2_]_*n*_ (**2**), were easily self-assembled and fully characterized. Both compounds represent the unique examples of silver(I) bioCPs driven by homophthalate^2–^ or 2,6-naphthalenedicarboxylate^2–^ linkers, thus widening a limited application of these dicarboxylate ligands for designing new metal–organic architectures.

The products **1** and **2** were applied as potent antimicrobial agents for the production of a new series of hybrid bioCP-loaded biopolymer films. Two types of biopolymer films, based on the epoxidized SBO or PS, were fabricated, and their antibacterial and biofilm inhibition properties were investigated in detail against four typical types of bacteria. In fact, the Ag-doped biopolymer films were able to prevent biofilm growth, especially those composed by [PS]_*n*_ for which a total biofilm inhibition was achieved in several cases. Interestingly, the efficacy of bioCPs was dependent on the biopolymer matrix, with **1** being more effective in [PS]_*n*_, while for **2**, this trend was less evident, exhibiting inhibition responses of the same magnitude. However, **2**@[PS]_*n*_ exhibited slightly better results against *P. aeruginosa* and *E. coli*, while **2**@[SBO]_*n*_ was more successful against *S. aureus* and *S. epidermidis*. The usage of two model biopolymers with different permeabilities and stabilities was one of the ideas explored in the present study to obtain materials with higher or lower Ag^+^ release, while applying the same type of dopant.

In summary, this work contributes to an underexplored biofilm inhibition application of bioCPs and derived functional materials, also showing that highly efficient antimicrobial biopolymer films can be easily fabricated from inexpensive biobased raw materials such as soybean oil, PS, and glycerol. We believe that this study can open up new perspectives for the design and application of biopolymer materials with antimicrobial activity.

## Supporting Information Available

Description of methods and experimental procedures and synthesis of bioCPs **1** and **2**, detailed photographs of powders and crystals of **1** and **2**, topological representations, photographs of [SBO]_*n*_ and [PS]_*n*_ biopolymer films, photographs of doped biopolymer films, particle size distribution analysis, ATR-FT-IR spectra, SEM-EDS data, images of biopolymer coupons in PBS solution, ICP-OES data, water absorption data, antibacterial properties of relevant Ag-based coordination polymers/MOFs, nonnormalized minimum inhibitory radius for different Ag-doped biopolymer films, TGA curves, PXRD patterns, additional antimicrobial data, and crystallographic data in CIF format (CCDC 2055525 and 2055526) (PDF)
