# Supplementary material for: Hybrid Silver(I)-Doped Soybean Oil and Potato Starch Biopolymer Films to Combat Bacterial Biofilms
Source: ACS Appl Mater Interfaces. 2022 May 27;14(22):25104–14. doi: 10.1021/acsami.2c03010 (PMC9773233; doi:10.1021/acsami.2c03010)
Supplement: Supplementary file 1 — am2c03010_si_001.pdf [file am2c03010_si_001.pdf]

Supporting Information (SI)

Hybrid Silver(I)-Doped Soybean Oil and Potato Starch Biopolymer Films to Combat Bacterial Biofilms

Tiago A. Fernandes,[†] Inês F.M. Costa,[†] Paula Jorge,[‡,δ] Ana Catarina Sousa,[†,§] Vânia André,[†] Rafaela G. Cabral,[†,§] Nuno Cerca,[‡,δ,*] Alexander M. Kirillov[†,*]

[†]*Centro de Química Estrutural, Institute of Molecular Sciences, Departamento de Engenharia Química, Instituto Superior Técnico, Universidade de Lisboa, Av. Rovisco Pais, 1049-001 Lisbon, Portugal. E-mail: kirillov@tecnico.ulisboa.pt*

[δ]*LABBELS–Associate Laboratory, Braga/Guimarães, Portugal*

[‡]*Centre of Biological Engineering, University of Minho, Campus de Gualtar, 4710-057 Braga, Portugal. E-mail: nunocerca@ceb.uminho.pt*

[§]*Área Departamental de Engenharia Química, ISEL - Instituto Superior de Engenharia de Lisboa, Instituto Politécnico de Lisboa, R. Conselheiro Emídio Navarro, 1, 1959-007 Lisbon, Portugal*

Supporting Information contains: Methods and experimental procedures, synthesis of bioCPs **1** and **2** (Fig. S1), detailed photographs of powders and crystals of **1** and **2** (Fig. S2), topological representations (Fig. S3), photographs of $[SBO]_n$ and $[PS]_n$ biopolymer films (Fig. S4), photographs of doped biopolymers films (Fig. S5), particle size distribution analysis (Fig. S6), ATR-FT-IR spectra (Figs. S7−S14), SEM-EDS data (Figs. S15 and S16), images of biopolymer coupons in PBS solution (Fig. S17), ICP-OES data (Fig. S18), water absorption data (Table S1), antibacterial properties of relevant Ag-based coordination polymers/MOFs (Table S2), nonnormalized minimum inhibitory radius for different Ag-doped biopolymer films (Table S3), TGA curves (Fig. S19), PXRD patterns (Figs. S20 and S21), additional antimicrobial data (Figs. S22−S28), and crystallographic data in CIF format (CCDC 2055525 and 2055526).

Materials and Methods. All chemicals and solvents were purchased from commercial sources. FTIR-ATR spectra were recorded on a Shimadzu IRAffinity-1S apparatus equipped with an ATR ZnSe Performance Crystal Plate accessory. Absorbance spectra were collected in the 4000–400 cm^{-1} range with a 2.0 cm^{-1} resolution using 64 co-added scans (abbreviations: vs − very strong, s − strong, m – medium, w – weak, br – broad, sh − shoulder). NMR spectra (^{1}H, ^{13}C) were obtained on a Bruker Avance II^{+} Spectrometer (400 MHz) using DMSO-d_6 (δ = 2.50 ppm) and TMS (δ = 0 ppm) as an internal reference. EA (elemental analyses) were run on a Perkin Elmer PE 2400 Series II analyzer by Laboratory of Analyses of IST. SEM and SEM-EDX was acquired using a Hitachi equipment, model S2400 with the accelerating voltage of 20.0 kV and equipped with a SDD EDS detector for light elements by Bruker. Particle size analysis of the prepared bioCPs was performed on Mastersizer 3000 (Malvern Panalytical) using samples dispersed in water. The determination of Ag ions was performed by ICP-OES (Perkin Elmer Optical Emission Spectrometer Optima 2000 DV) with the following operation conditions: RF power 1300 W, auxiliary gas flow 0.2 $L{\cdot}min^{-1}$, nebulizer gas flow 0.6 $L{\cdot}min^{-1}$, coolant gas flow 15 $L{\cdot}min^{-1}$, Sample Flow Rate 1.50 $mL{\cdot}min^{-1}$, and Ag analytical line 328.068 nm (LAIST, Laboratory of Analyses, IST).

Thermogravimetric Analysis (TGA). Thermal stability of doped and undoped $[SBO]_n$ and $[PS]_n$ samples were evaluated using a Perkin Elmer STA 6000 simultaneous thermal analyser (Waltham, MA, USA), using dried samples (~10 mg, N_2 flow, 10 °C min^{-1} heating (Fig. S19).

Powder X-Ray Diffraction (PXRD). Data were collected on a D8 Advance Bruker AXS θ-2θ diffractometer, equipped with a LYNXEYE-XE detector, copper radiation source (Cu Kα, λ=1.5406 Å), operated at 40 kV and 30 mA, with a Ni filter and using the following data collection parameters: 3-60° 2θ range, step size of 0.02° and 57.6 s per step. The diffractograms were used to confirm the purity of microcrystalline samples, by comparing the calculated (from single-crystal X-ray diffraction data) and experimental PXRD patterns. MERCURY 2020.3.0[1] was used for calculating the patterns from the crystal structures (Figs. S20, S21).

Single crystal X-ray Diffraction. Single crystals of **1** and **2** suitable for X-ray diffraction were mounted with Fomblin© in a cryoloop. Diffraction data were collected on Bruker AXS-KAPPA APEX II or BRUKER D8 QUEST diffractometers (graphite-

monochromated radiation, Mo Kα, λ = 0.7107 Å, 298 K). X-ray generator parameters were 50 kV and 30 mA. APEX2 and APEX3 programs were used to monitor the collection of X-ray data. SAINT and SADABS were applied for correction of all data for Lorentzian, polarization, and absorption effects. SHELXT was used for structure solution, while SHELXL-97 was applied for full matrix least-squares refinement on F^2.[2] These programs are included in the package of programs, WINGX-Version 2014.1.[3] Non-H atoms were refined anisotropically. Full-matrix least-squares refinement was used for the non-hydrogen atoms with anisotropic thermal parameters. All hydrogen atoms bonded to carbon atoms were geometrically placed and refined in the parent carbon atoms. Water hydrogen atoms were inserted while considering the electron density map and the distances were restrained. In **2**, the Ag atom is disordered over two positions with 0.87 and 0.13 occupancy. Crystal data are summarized in Table 1. CCDC 2055525 and 2055526.

Bacteria and Growth Conditions. Two Gram negative (*P. aeruginosa* PA14 and *E. coli* ATCC 25922) and two Gram positive (*S. aureus* ATCC 25923 and *S. epidermidis* RP62A) biofilm forming bacteria (collection of the Centre of Biological Engineering (CEB), University of Minho) were used in this study. Bacteria were grown at 37 ^{o}C aerobically and under agitation (120 rpm). Standard growth media was used, namely Mueller-Hinton broth (MHB) (Liofilchem) and Mueller-Hinton agar (MHA) (Liofilchem) in antibacterial assays and tryptic soy broth (TSB) (Liofilchem) and tryptic soy agar (TSA) (Liofilchem) in biofilm inhibition assays. A 0.9% sodium chloride (NaCl) (AnalaR NORMAPUR) solution was used for washing and diluting the samples.

Antibacterial and Biofilm Inhibition Activity Studies. Compounds **1** and **2** and their respective doped films were assessed in a soft agar overlay assay for their antibacterial activity. Briefly, bacteria were grown overnight in MHB and then transferred to warm soft MHA (0.5% agar) at a final concentration of 1 × 10^6 colony forming units (CFU) mL^{-1}. 3 mL of inoculated soft MHA were placed on top of 10 mL of solidified hard MHA (1.7% agar) in a 9 cm Petri dish. Both the bioCPs (≈ 4 mg each) and their respective doped films (cut to 0.9 cm of diameter round discs) were placed on top of the soft MHA and incubated for 24 h. Bacterial growth inhibition, indicated by a "no grow" halo around the samples, was analyzed by photographing the plates in a Bio-Rad ChemiDoc™ Imager and measuring the minimum inhibition radius for bioCPs and films, respectively, with the help of the image editing software GIMP.

In turn, biofilm inhibition activity was assessed by growing bacteria in the presence of the films to check for biofilm formation on their surface. Briefly, bacteria were grown overnight in TSB and then transferred to fresh media at a final concentration of 1×10^6 CFU mL^{-1}. The films were placed into a 48 (for $[SBO]_n$) or 24 (for $[PS]_n$) well microtiter plate, submerged in 300 µL (for $[SBO]_n$) or 1000 µL (for $[PS]_n$) of bacterial suspension, and incubated for 24 h. The films were removed, placed in clean wells, and washed twice to remove non-adhered cells. The adhered bacteria were detached in 300 µL (for $[SBO]_n$) or 1000 µL (for $[PS]_n$) of NaCl solution in an ultrasonic bath (220 V, 50/60 Hz) for 15 min followed by 30 s vortex. Detached cells were quantified by serially diluting the samples, plating them intro TSA, and counting the CFU after overnight growth. Biofilm formation was presented as the logarithm of the amount of detached cells per cm^2 of film. The different methodology used for the two films was due to the swelling of the $[PS]_n$ films in contact with liquid media which made them unfit for small wells.

All experiments were performed at least three times and data was analyzed using Excel and GraphPad software. For comparison, the obtained antibacterial and biofilm inhibition activity data for **1**, **2**, Ag_2O (or $AgNO_3$) and the respective films were normalized for the molar content of silver(I) in each sample. The nonnormalized data are given in Figs. S22−S28.

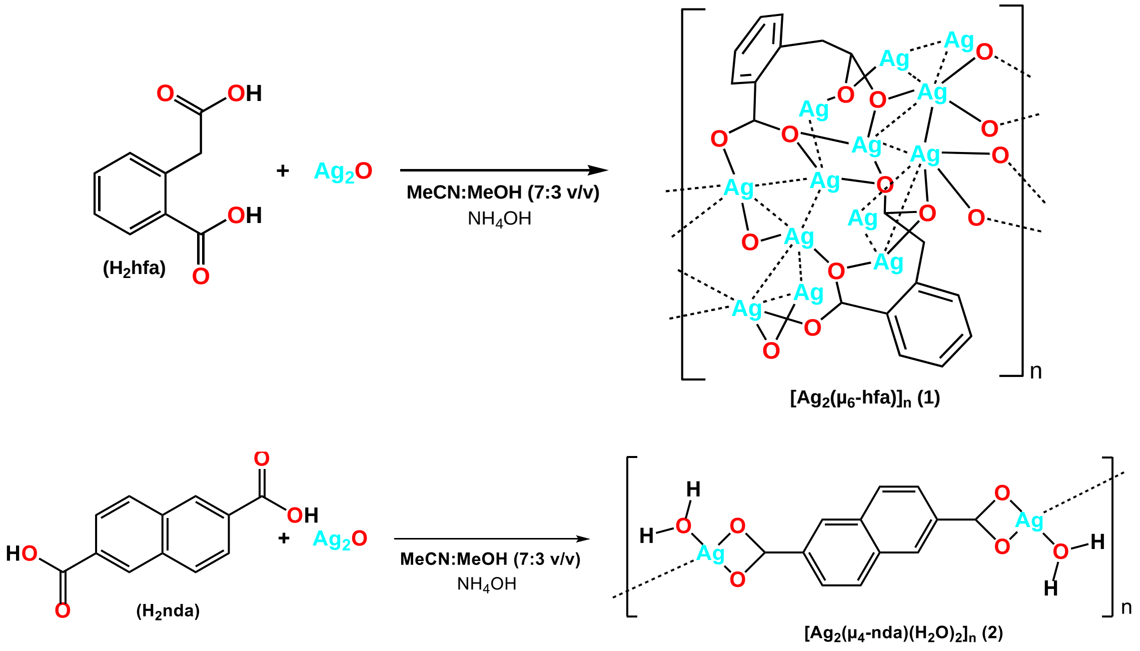

Figure S1. Synthesis of bioCPs **1** and **2**.

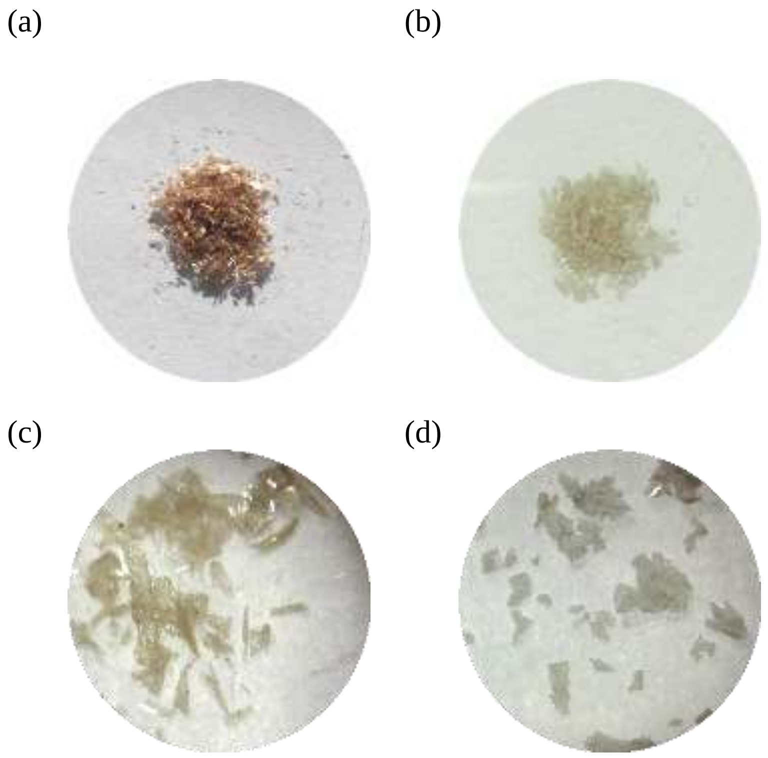

Figure S2. (a, b) Photographs of (a) $[Ag_2(\mu_6\text{-hfa})]_n$ (**1**) and (b) $[Ag_2(\mu_4\text{-nda})(H_2O)_2]_n$ (**2**) solids. (c, d) Photographs of crystalline powders of (c) **1** and (d) **2** at 4× magnification.

Topological Features of 1 and 2. From a topological standpoint, a simplified double layer in **1** is constructed from the 3-connected Ag1/Ag2 centers and the 6-connected μ_6-hfa^{2-} nodes (Fig. S3a,b). The resulting binodal 3,6-connected layer features a 3,6L64 topology with a $(4^2.6)_2(4^4.6^9.8^2)$ point symbol, wherein the $(4^2.6)$ and $(4^4.6^9.8^2)$ indices correspond to the Ag and μ_6-hfa^{2-} nodes, respectively. A simplified supramolecular net of **2** can be described as a mononodal 4-connected layer with an sql topology and a $(4^4.6^2)$ point symbol (Fig. S3c).

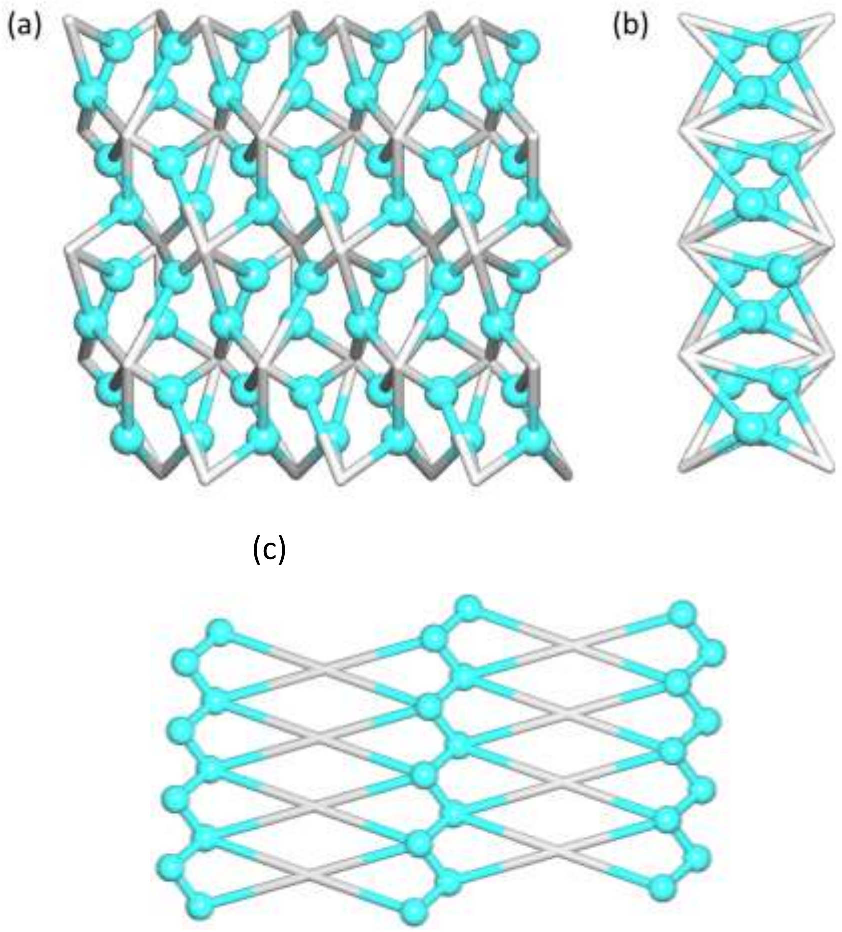

Figure S3. (a,b) Topological views of a binodal 3,6-connected metal-organic layer in **1** with a 3,6L64 topology; 3-linked Ag nodes (cyan balls), centroids of 6-linked μ_6-hfa^{2-} nodes (gray); views along the *c* (a) and *a* (b) axis. (c) Topological view of a mononodal 4-connected supramolecular net in **2** with an sql topology; 4-linked Ag nodes (cyan balls), centroids of 4-linked μ_4-nda^{2-} nodes (gray); view along the *c* axis.

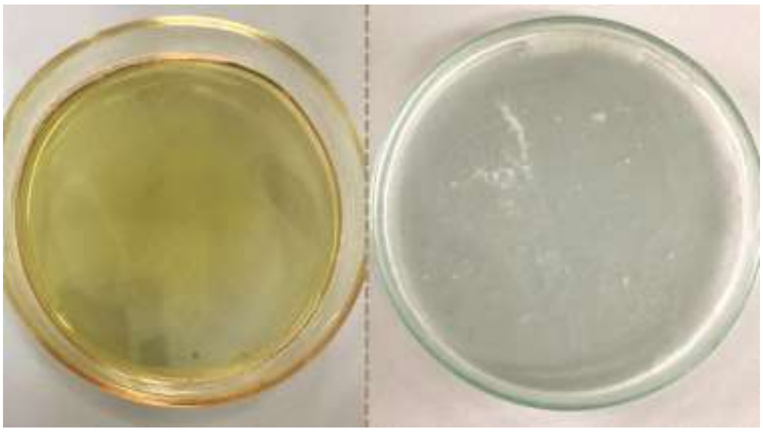

Figure S4. Photographs of as-made $[SBO]_n$ and $[PS]_n$ biopolymer films in Petri dishes.

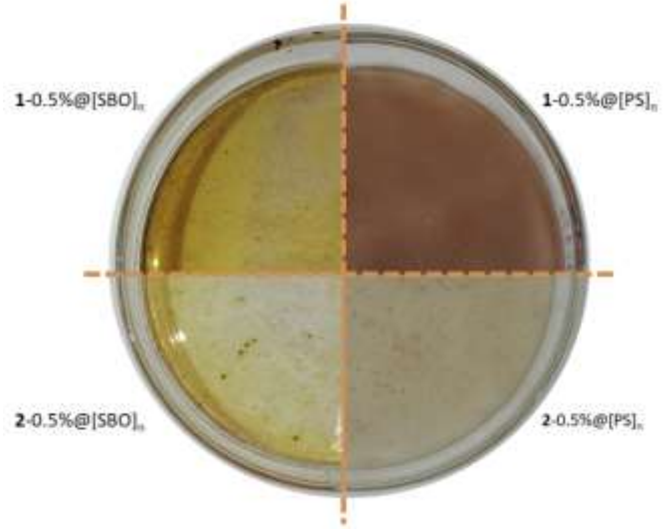

Figure S5. Photographs of as-made Ag-doped $[SBO]_n$ and $[PS]_n$ biopolymer films in Petri dishes.

(a)

(b)

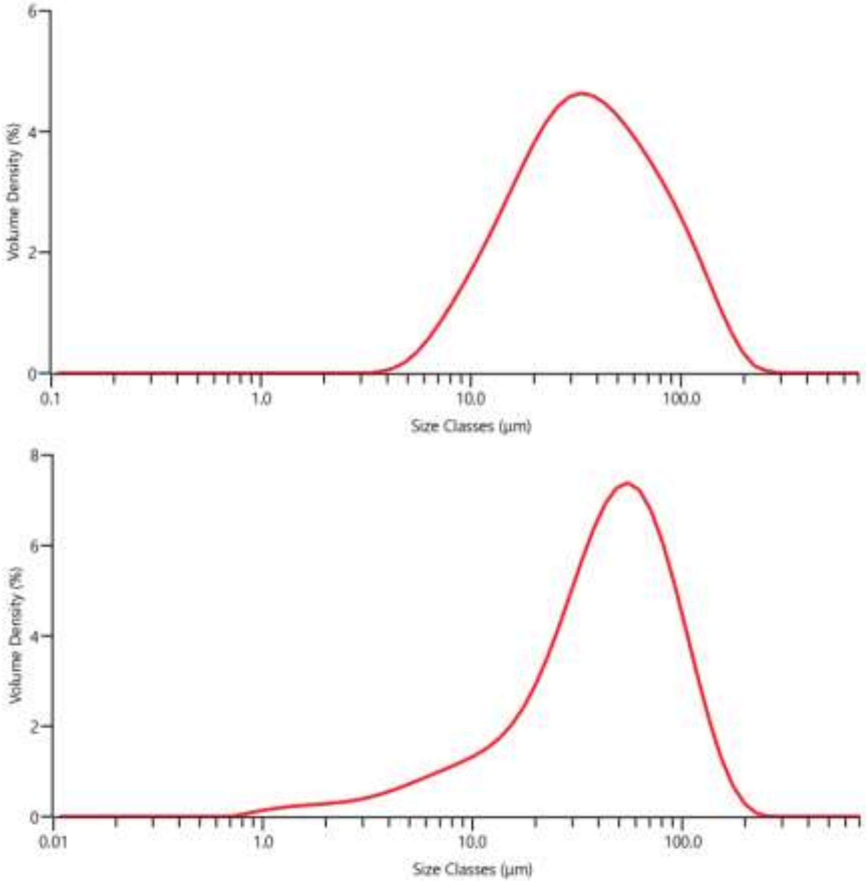

Figure S6. Particle size distribution plots for the samples of **1** (a) and **2** (b) dispersed in water (mean particle size of 66 and 51 μm, respectively).

.

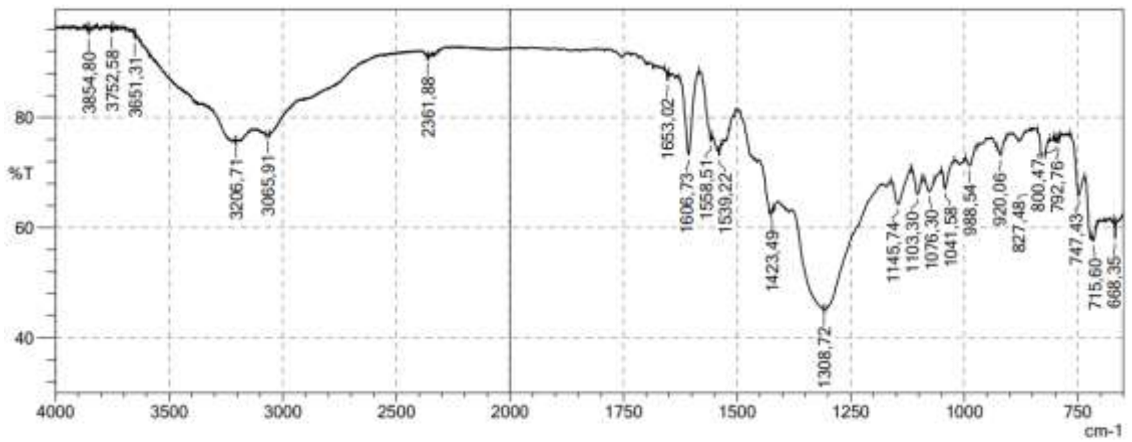

Figure S7. FTIR spectrum of **1**.

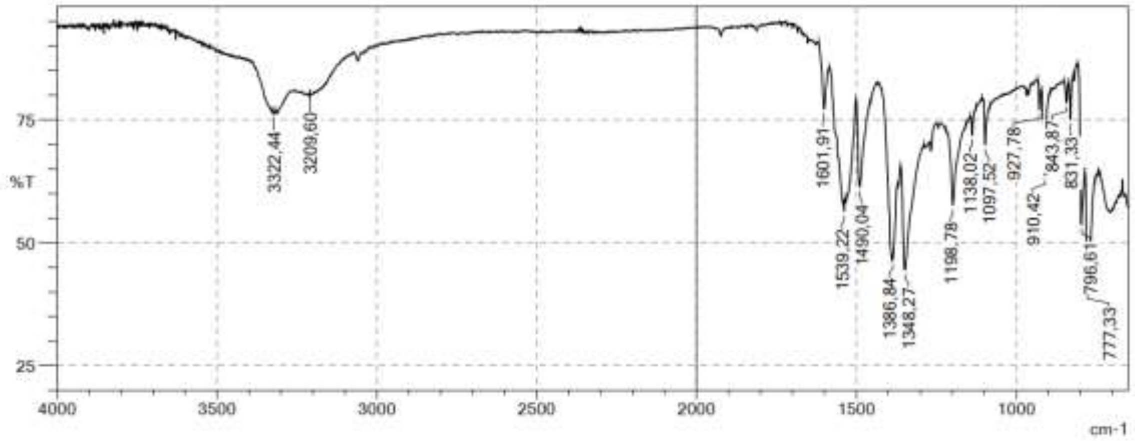

Figure S8. FTIR spectrum of **2**.

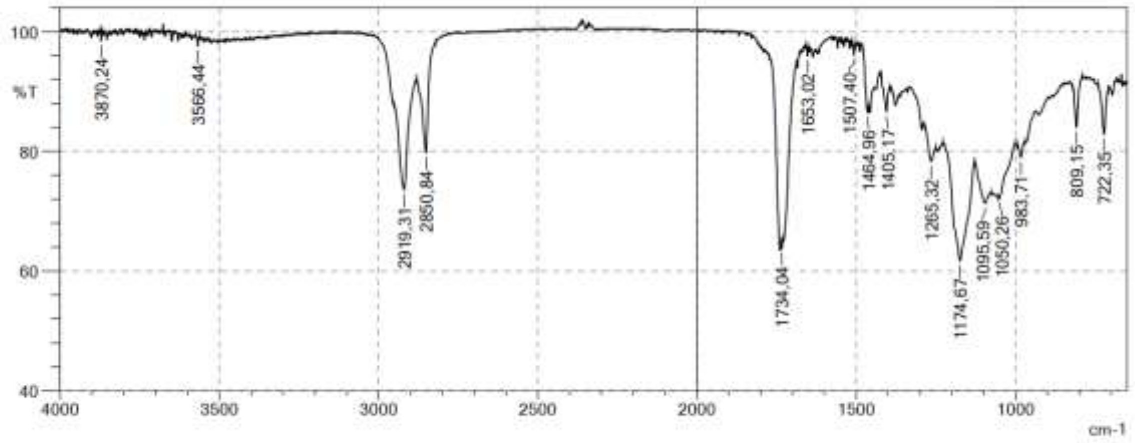

Figure S9. FTIR spectrum of $[SBO]_n$.

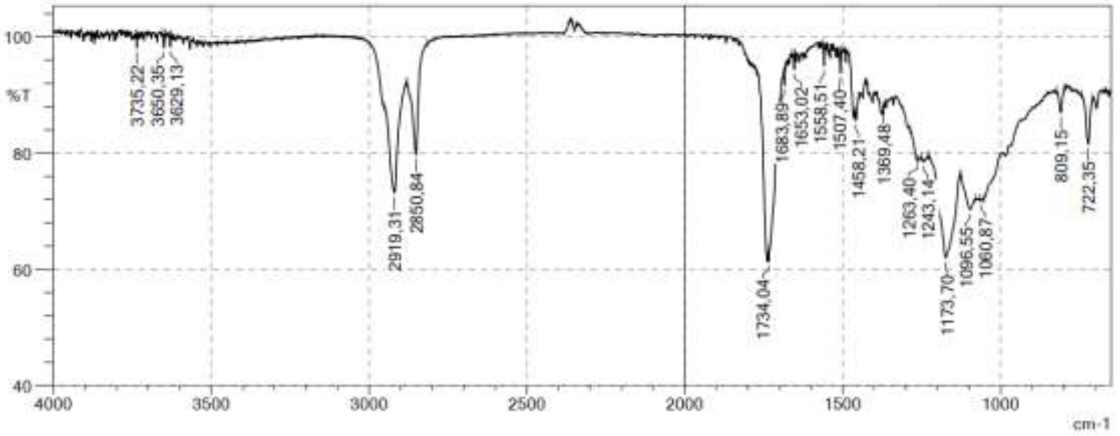

Figure S10. FTIR spectrum of **1-**0.5%@[SBO]$_n$.

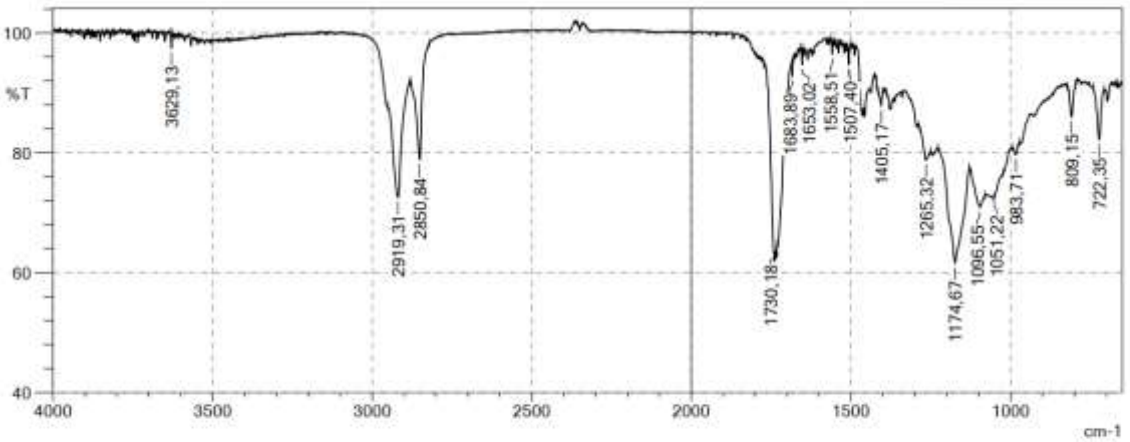

Figure S11. FTIR spectrum of **2-**0.5%@[SBO]$_n$.

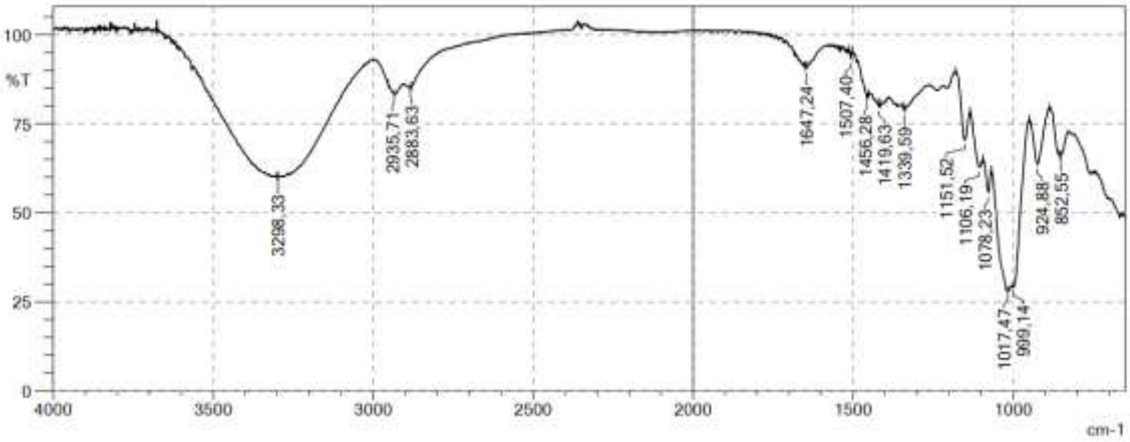

Figure S12. FTIR spectrum of [PS]$_n$.

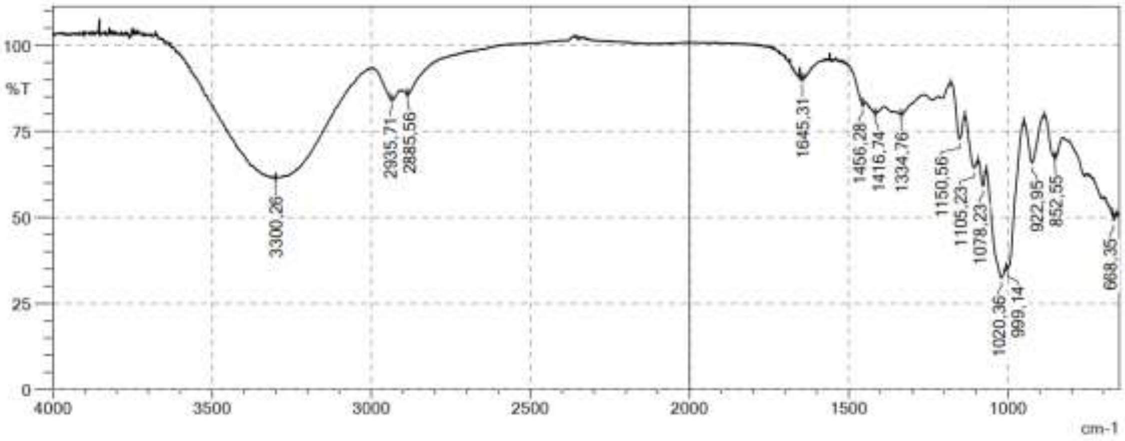

Figure S13. FTIR spectrum of **1**-0.5%@$[PS]_n$.

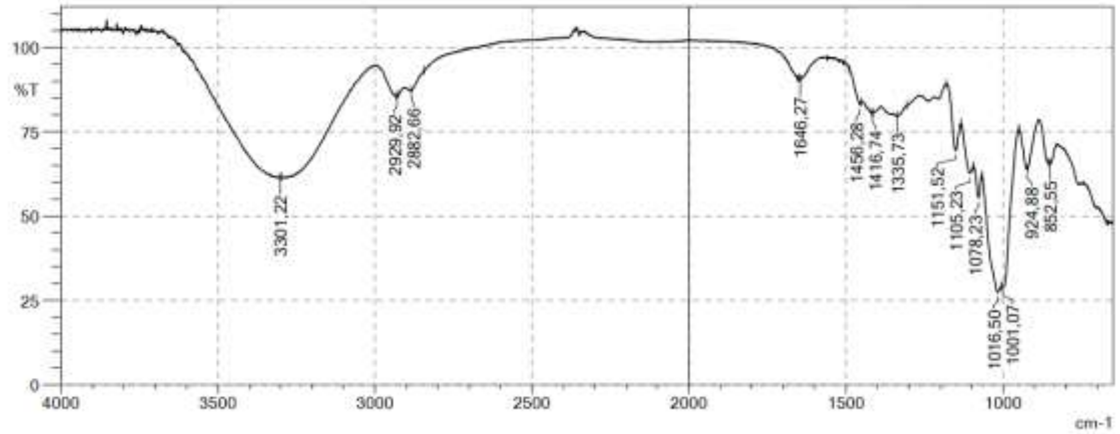

Figure S14. FTIR spectrum of **2**-0.5%@$[PS]_n$.

Discussion of FTIR spectra. The FTIR spectra of **1** and **2** reveal the characteristic intense bands at 1594−1583 and 1358−1352 cm^{-1} due to asymmetric and symmetric vibrations of carboxylate groups, respectively.

The ATR-FTIR spectra of $[SBO]_n$ and doped bioCP@$[SBO]_n$ and Ag_2O@$[SBO]_n$ films show no significant differences, reflecting the $[SBO]_n$ profile with intensive absorption bands at 1737 and 1166 cm^{-1} due to the stretching vibrations of C=O and ether C–O–C functionalities, respectively. The aliphatic sequence was also identified by the presence of the intensive bands at 2918 and 2849 cm^{-1} consistent with the asymmetric and symmetric stretching vibrations of C–H groups.

The ATR-FTIR spectra of $[PS]_n$ show intervention of the glycerol plasticizer with potato starch. This resulted in a shift of the O–H and C–H bands suggesting a substitution of hydroxyl bonds between the starch polymer chains by weaker hydrogen bonds between the starch and the plasticizer. The presence of a broad $\nu(OH/H_2O)$ band around 3300 cm^{-1} is one of the most distinctive features of the $[PS]_n$ films in comparison to $[SBO]_n$. Doped bioCPs@$[PS]_n$ films show no significant differences, reflecting the profile of $[PS]_n$ spectrum with characteristic peaks for

potato starch. All samples reveal the same characteristic peaks at 3299 cm^{-1} ν(OH/H_2O), 2936 cm^{-1} ν(CH), and 1647 cm^{-1} δ(H_2O).[4,5]

In the fingerprint region, it is possible to observe low intensity vibration bands between 1460 and 1340 cm^{-1} assigned to σ(CH_2), σ(CH), both of which are assigned as modes involving the deformation of the CH_2 group, and which also comprehend the σ(OH), at 1339 cm^{-1}.[1,2,6]

There are also intensive and broad bands at 1017 cm^{-1} assigned to the crystalline and amorphous regions of starch that correspond to ν(C–O), ν(C–C), or σ(C–OH).[2] Vibrations of the glucosidic C–O–C bond and the entire glucose ring are associated with a band at 1151 cm^{-1}; herein diverse modes of vibration and bending can be invoked, i.e. ν(C–O), ν(C–O–H), or σ(C–H).[1] The band at 925 cm^{-1} can be assigned to the skeletal mode of the C–C stretch and the skeletal modes of the pyranose ring, and skeletal mode of α(1→4) glycosidic linkage.[7]

In both cases it is not possible to differentiate by FTIR pure biopolymers $[SBO]_n$ or $[PS]_n$ and their bioCP-doped analogues due to a very low loading of the latter (max. 0.5%).

(a)

(b)

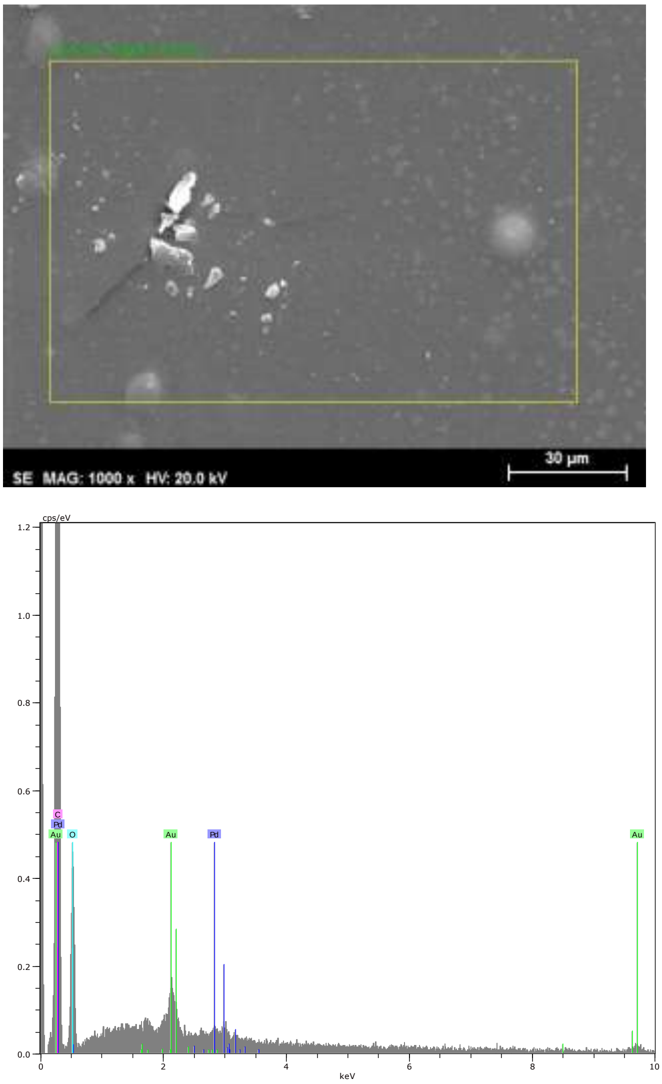

Figure S15. (a) SEM image at 150× magnification of **1**-0.5%@[SBO]$_n$ biopolymer film with (b) EDS analysis of elemental composition.

(a)

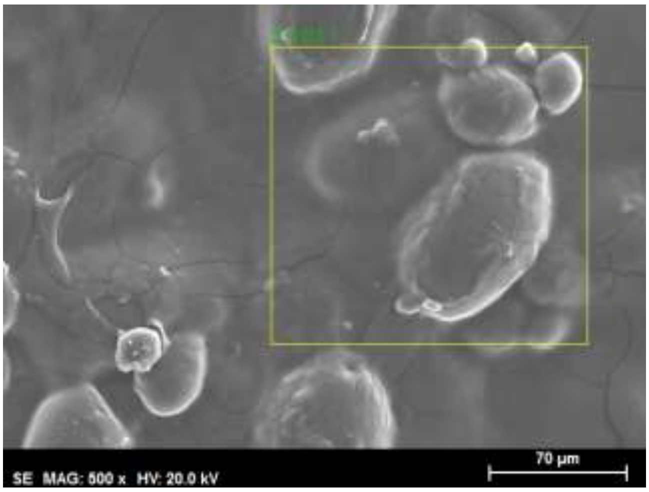

(b)

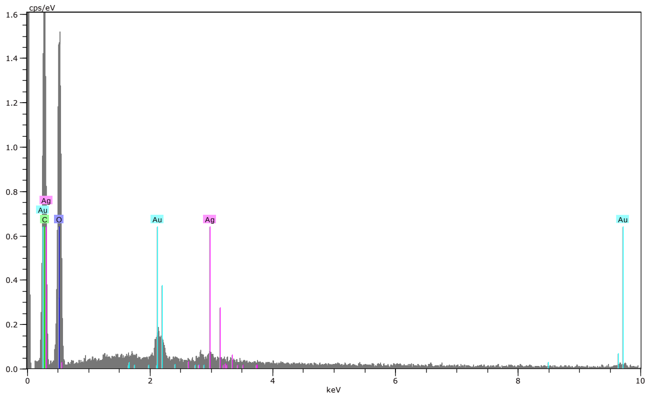

Figure S16. (a) SEM image at 150× magnification of **1-**0.5%@[PS]$_n$ biopolymer film with (b) EDS analysis of elemental composition.

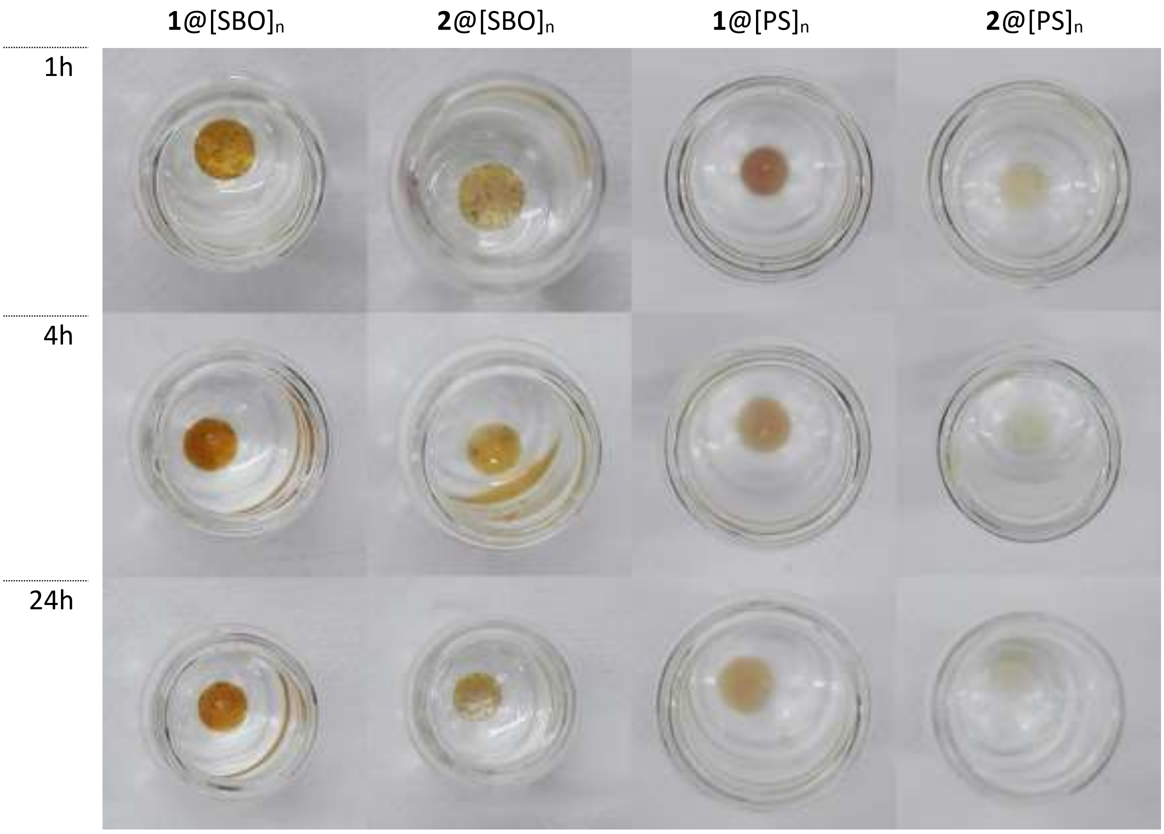

Figure S17. Images of hybrid biopolymer films **1**-0.5%@[SBO]$_n$, **2**-0.5%@[SBO]$_n$, **1**-0.5%@[PS]$_n$, and **2**-0.5%@[PS]$_n$ in aqueous PBS solution over time. Visible physical changes are noticed only for **1**-0.5%@[SBO] and **1**-0.5%@[PS]$_n$, that became clearer over time. Solution samples (1 mL) were submitted to ICP-OES analysis to monitor silver release.

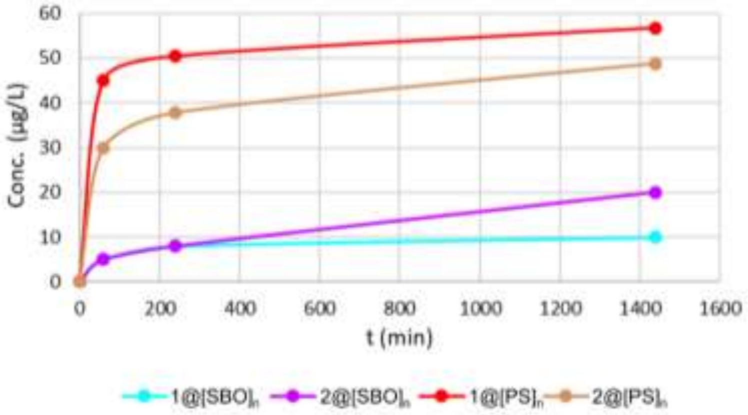

Figure S18. Silver ion release from **1**-0.5%@[SBO]$_n$, **2**-0.5%@[SBO]$_n$, **1**-0.5%@[PS]$_n$, and **2**-0.5%@[PS]$_n$ after 1, 4, and 24 h in aqueous Phosphate Buffered Saline solution (within 24 h, the Ag^+ release of 10 µg/L corresponds to 0.06% of all silver present in **1**@[SBO]$_n$; Ag^+ release of 20 µg/L corresponds to 0.11% of all silver present in **2**@[SBO]$_n$; Ag^+ release of 57 µg/L corresponds to 0.36% of all silver present in **1**@[PS]$_n$; Ag^+ release of 49 µg/L corresponds to 0.27% of all silver present in **2**@[PS]$_n$).

Water Absorption by Biopolymers. In order to investigate water absorption by the obtained samples, the biopolymer films (0.06-0.21 mg) were submerged in water (20 mL; ~25 °C), and their mass was determined after 6 and 24 h.

$$Wup\ (\%) = \frac{W_f - W_i}{W_i} \times 100 \qquad (1)$$

where, W_i and W_f are the initial and final weights of the samples, respectively.

Table S1. Water absorption determination through mass weighing for $[SBO]_n$ and $[PS]_n$ biopolymer films.

Biopolymer	Sample	W_i (mg)[a]	W_f (mg)[b]		W_{up} (%)[c]		Average W_{up} (%)
		0 h	6 h	24 h	6 h	24 h	24 h
$[SBO]_n$	a	0.213	0.216	0.216	1.39	1.39	1.5
	b	0.172	0.174	0.175	1.15	1.71	
	c	0.156	0.158	0.159	1.27	1.89	
$[PS]_n$	a	0.06	0.151	0.15	60.26	60.00	59.4
	b	0.06	0.148	0.147	59.46	59.18	
	c	0.059	0.143	0.142	58.74	58.45	

[a]Starting weight. [b]Weight after 6 and 24 h. [c]Water uptake weight percentage after 6 and 24 h.

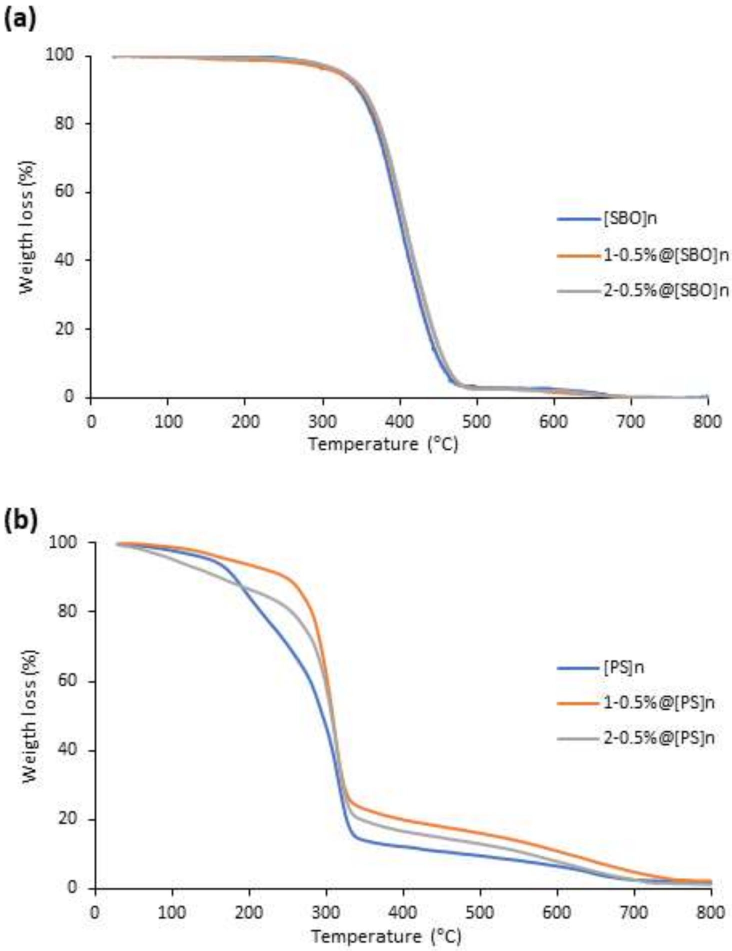

Figure S19. Thermogravimetric analysis curves for (a) $[SBO]_n$, **1**-0.5%@$[SBO]_n$, **2**-0.5%@$[SBO]_n$ and (b) $[PS]_n$, **1**-0.5%@$[PS]_n$, **2**-0.5%@$[PS]_n$ biopolymer films.

Table S2. Antibacterial Properties of Relevant Ag-Based Coordination Polymers/MOFs.

#	Formula	nD	Silver environment	Ligand	Type of bacteria	Antibacterial activity	Reference
**1**	$[Ag(\mu\text{-ebin})_2)NO_3]_n$	1D	Ag-O	ethanediyl bis(isonicotinate) (ebin)	*S. epidermidis* *S. aureus*	biofilm formation prevention; prevention of murine S. epidermidis implant infection	Gordon *et al.*, 2010 [57]
**2**	$[Ag_3(\mu_3\text{-ppb})]_n$	3D	Ag-O Ag-P	3-phosphonobenzoate (H_3ppb)	*E. coli* *P. aeruginosa* *S. aureus*	active against 6 different bacterial strains	Berchel *et al.* 2011 [58]
**4**	$[Ag_2(\mu_2\text{-PTA})(\mu_3\text{-PTA})(\mu_2\text{-pga})(H_2O)]_n\cdot 6H_2O$	2D	Ag-O Ag-N Ag-P	1,3,5-triaza-7-phosphaadamantane (PTA) and 3-phenylglutaric acid (H_2pga)	*E. coli* *P. aeruginosa* *S. aureus*	antibacterial and antifungal activity	Jaros *et al.*, 2016 [18]
**5**	$[Ag_2(\mu_2\text{-PTA})(\mu_3\text{-PTA})(Hpmal)_2]_n\cdot 2H_2O$	1D	Ag-O Ag-N Ag-P	PTA and phenylmalonic acid (H_2pmal)	*E. coli* *P. aeruginosa* *S. aureus*		
**6**	$[Ag(\mu_3\text{-PTA})(Hdmga)]_n$	2D	Ag-O Ag-N Ag-P	PTA and 3,3-dimethylglutaric acid (H_2dmga)	*E. coli* *P. aeruginosa* *S. aureus*		
**7**	$[Ag(\mu_3\text{-PTA})(chc)]_n\cdot n(Hchc)\cdot 2nH_2O$	2D	Ag-O Ag-N Ag-P	PTA and cyclohexanecarboxylic acid (Hchc)	*E. coli* *P. aeruginosa* *S. aureus*	efficient antibacterial activity at low concentrations	Jaros *et al.*, 2016 [19]
**8**	$[Ag_2(\mu_3\text{- PTA})_2(\mu_2\text{-chdc})]_n$	3D	Ag-O Ag-N Ag-P	PTA and 1,4-cyclohexanedicarboxylic acid (H_2chdc)	*E. coli* *P. aeruginosa* *S. aureus*		
**9**	$[Ag_2(\mu_2\text{-PTA})_2(\mu_4\text{-}H_2chtc)]_n\cdot 6nH_2O$	2D	Ag-O Ag-N Ag-P	PTA and 1,2,4,5-cyclohexanetetra-carboxylic acid (H_4chtc)	*E. coli* *P. aeruginosa* *S. aureus*		
**10**	$[Ag(\mu\text{-bib})]_n{}^{a}$	1D	Ag-N	1,3-bisimidazolyl benzene (bib)	*E. coli biofilms* *S. aureus*	contact-killing effect on amoxicillin-resistant E. coli biofilms	Wu *et al.*, 2018 [59]
**11**	$[Ag_4(\mu_8\text{-}H_2pma)_2]_n\cdot 4nH_2O^{b}$	3D	Ag-O	pyromellitic acid (H_4pma)	*S. epidermidis, S. aureus, P. aeruginosa and E. coli*	efficient antibacterial activity at low concentrations and anti-biofilm	Fernandes *et al.* 2021 [21]
**12**	$[Ag_5(\mu_8\text{-}H_{0.5}tma)_2(H_2O)_4]_n\cdot 2nH_2O^{b}$	3D	Ag-O	trimesic acid (H_3tma)	*S. epidermidis, S. aureus, P. aeruginosa and E. coli*		
**13**	$[Ag_2(\mu_6\text{-hfa})]_n{}^{c}$	2D	Ag-O	homophthalic acid (H_2hfa)	*S. epidermidis,*	efficient antibacterial activity at low concentrations and anti-biofilm	Present study
**14**	$Ag_2(\mu_4\text{-nda})(H_2O)_2]_n{}^{c}$	2D	Ag-O	2,6-naphthalenedicarboxylic acid (H_2nda)	*S. epidermidis, S. aureus, P. aeruginosa and E. coli*		

[a]Compound also supported on PEG1000, PEG220 or PEG550. [b]Compounds also supported on SBO. [c]Compounds also supported on SBO and PS.

Table S3. Normalized Minimum Inhibitory Radius for Different Ag-doped Biopolymer Films.

**Biopolymer film**	**Concentration of Ag-containing dopant (% m/m)**	**NORMALIZED DATA: Minimum Inhibitory Radius (mm)**			
		***P. aeruginosa* PA14**	***E. coli* ATCC 25922**	***S. aureus* ATCC 25923**	***S. epidermidis* RP62A**
$AgNO_3$@[SBO]$_n$	0.05	0.40 ± 0.15	0.00 ± 0.00	0.93 ± 0.73	0.18 ± 0.13
	0.1	1.55 ± 1.29	0.08 ± 0.06	0.87 ± 1.23	1.05 ± 0.94
	0.5	5.02 ± 1.61	4.08 ± 0.23	5.85 ± 1.37	5.53 ± 1.81
Ag_2O@[SBO]$_n$	0.05	0.00 ± 0.00	0.00 ± 0.00	0.00 ± 0.00	0.09 ± 0.06
	0.1	0.07 ± 0.10	0.00 ± 0.00	0.00 ± 0.00	0.28 ± 0.13
	0.5	0.39 ± 0.22	0.04 ± 0.06	0.26 ± 0.04	0.90 ± 0.22
[Ag_2(μ_6-hfa)]$_n$@[SBO]$_n$ (**1**@[SBO]$_n$)	0.05	0.00 ± 0.00	0.00 ± 0.00	0.00 ± 0.00	0.06 ± 0.08
	0.1	0.00 ± 0.00	0.00 ± 0.00	0.00 ± 0.00	0.21 ± 0.18
	0.5	0.62 ± 0.44	0.00 ± 0.00	0.03 ± 0.04	1.00 ± 0.44
[Ag_2(μ_4-nda)(H_2O)$_2$]$_n$@[SBO]$_n$ (**2**@[SBO]$_n$)	0.05	0.15 ± 0.12	0.00 ± 0.00	0.17 ± 0.15	0.64 ± 0.13
	0.1	2.28 ± 0.52	0.92 ± 0.27	2.70 ± 0.21	3.64 ± 0.22
	0.5	4.04 ± 0.28	1.73 ± 0.40	3.99 ± 0.19	5.89 ± 0.52

Data is shown as mean ± SD and were normalized for the molar content of silver(I) in each sample.

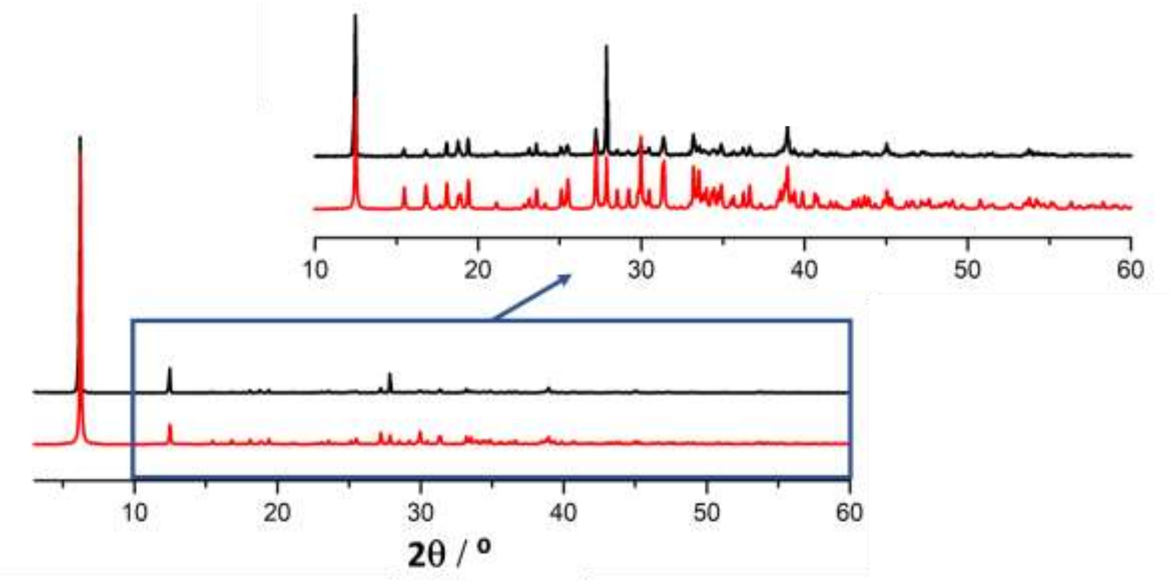

Figure S20. Experimental (black) and theoretical (red) powder diffraction patterns for **1**. The inset on top shows the peaks in the 10-60 2θ / ° range in different *y* scale for better visualization.

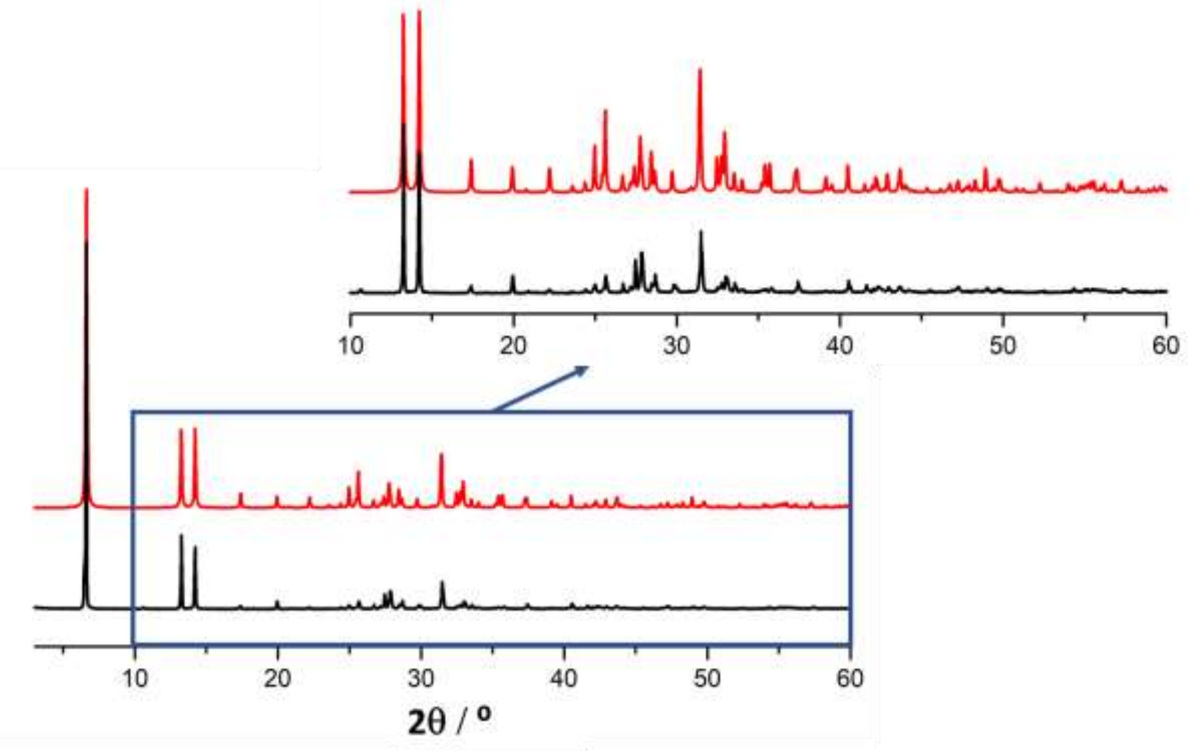

Figure S21. Experimental (red) and theoretical (black) powder diffraction patterns for **2**. The inset on top shows the peaks in the 10-60 2θ / ° range in different *y* scale for better visualization.

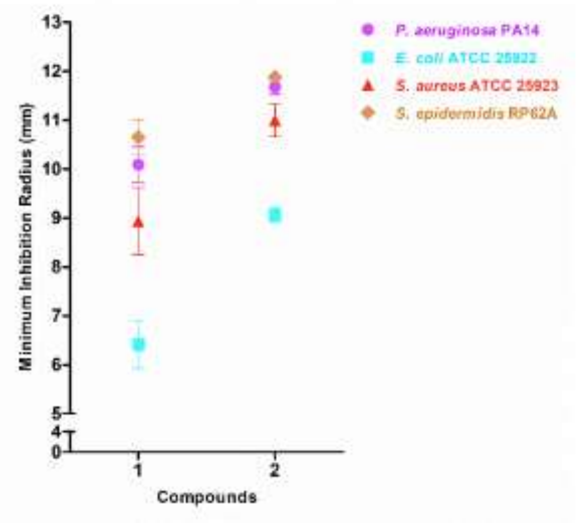

Figure S22. Nonnormalized antibacterial activity of bioCPs **1** and **2** against Gram negative (*P. aeruginosa* and *E. coli*) and Gram positive (*S. aureus* and *S. epidermidis*) bacteria.

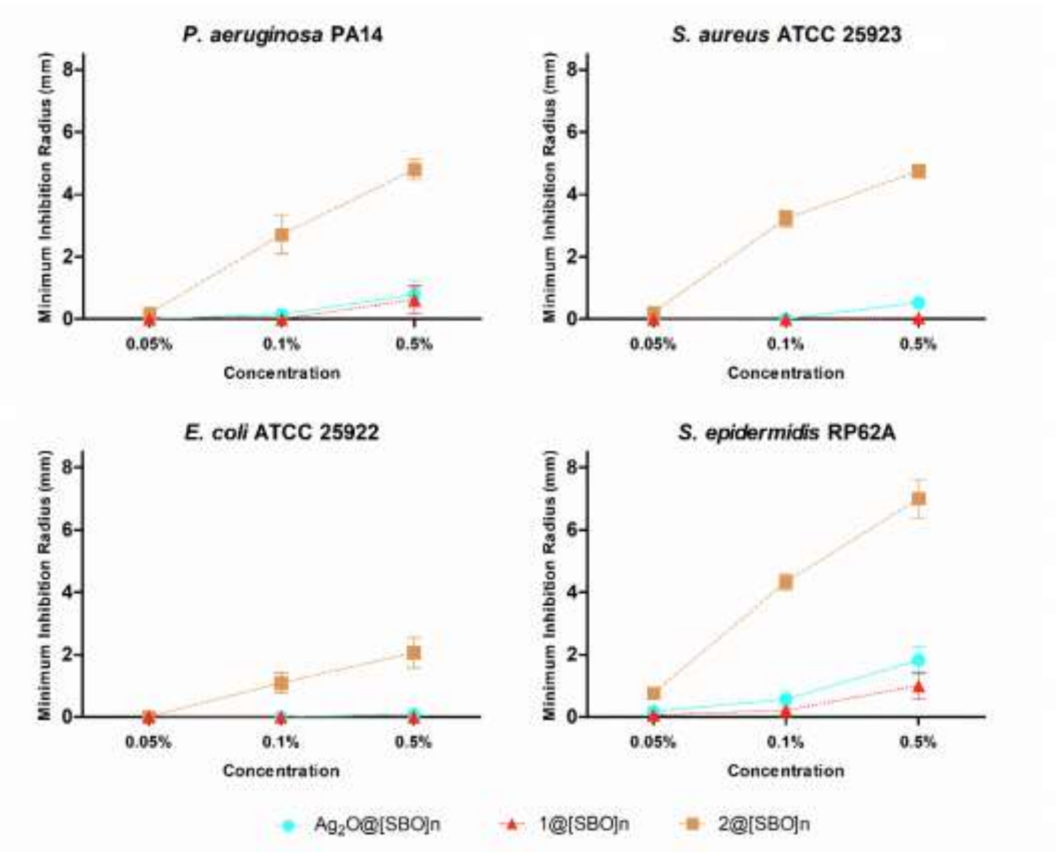

Figure S23. Nonnormalized antibacterial activity data of $[SBO]_n$ films doped with different concentrations (0.05, 0.1, and 0.5%) of Ag_2O ($Ag_2O@[SBO]_n$) and compounds **1** (**1**$@[SBO]_n$) and **2** (**2**$@[SBO]_n$) against Gram negative (*P. aeruginosa* and *E. coli*) and Gram positive (*S. aureus* and *S. epidermidis*) bacteria.

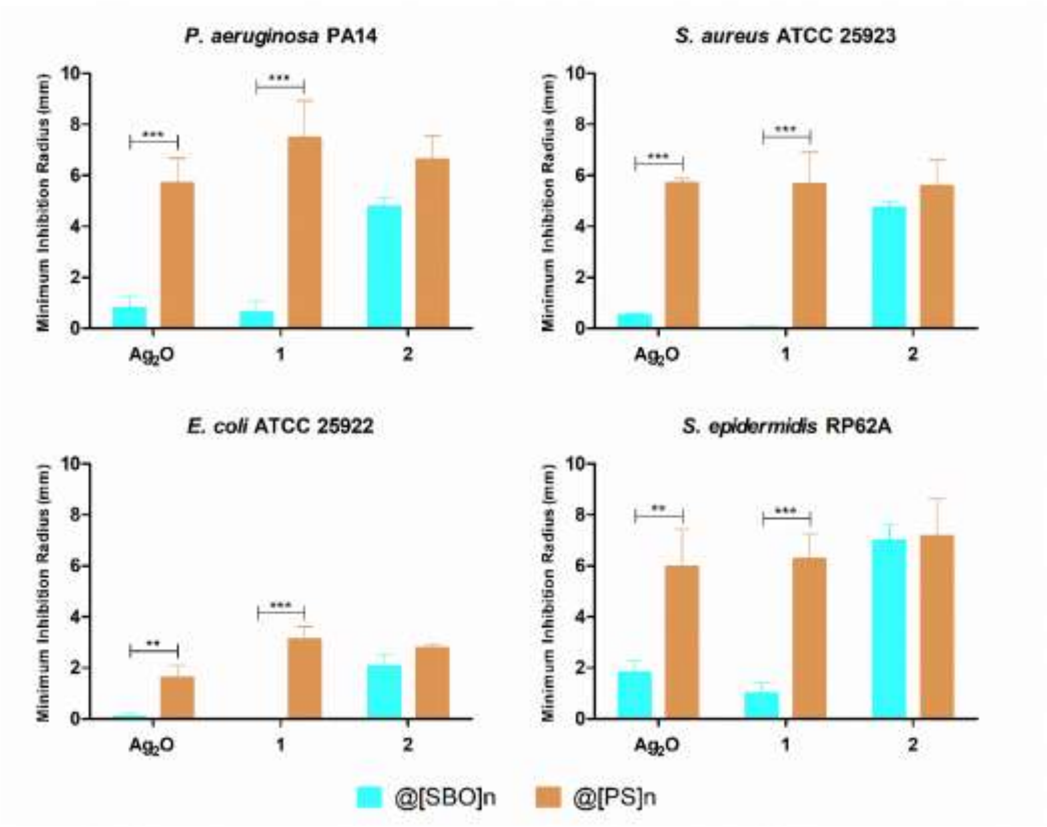

Figure S24. Nonnormalized antibacterial activity of $[SBO]_n$ *vs* $[PS]_n$ films doped with 0.5% of Ag_2O and compounds **1** and **2** against Gram negative (*P. aeruginosa* and *E. coli*) and Gram positive (*S. aureus* and *S. epidermidis*) bacteria. Significant statistical differences were found using the Bonferroni's Multiple Comparison Test with 95% confidence interval and are depicted as follows: * - $P \leq 0.05$; ** - $P \leq 0.01$; *** - $P \leq 0.001$.

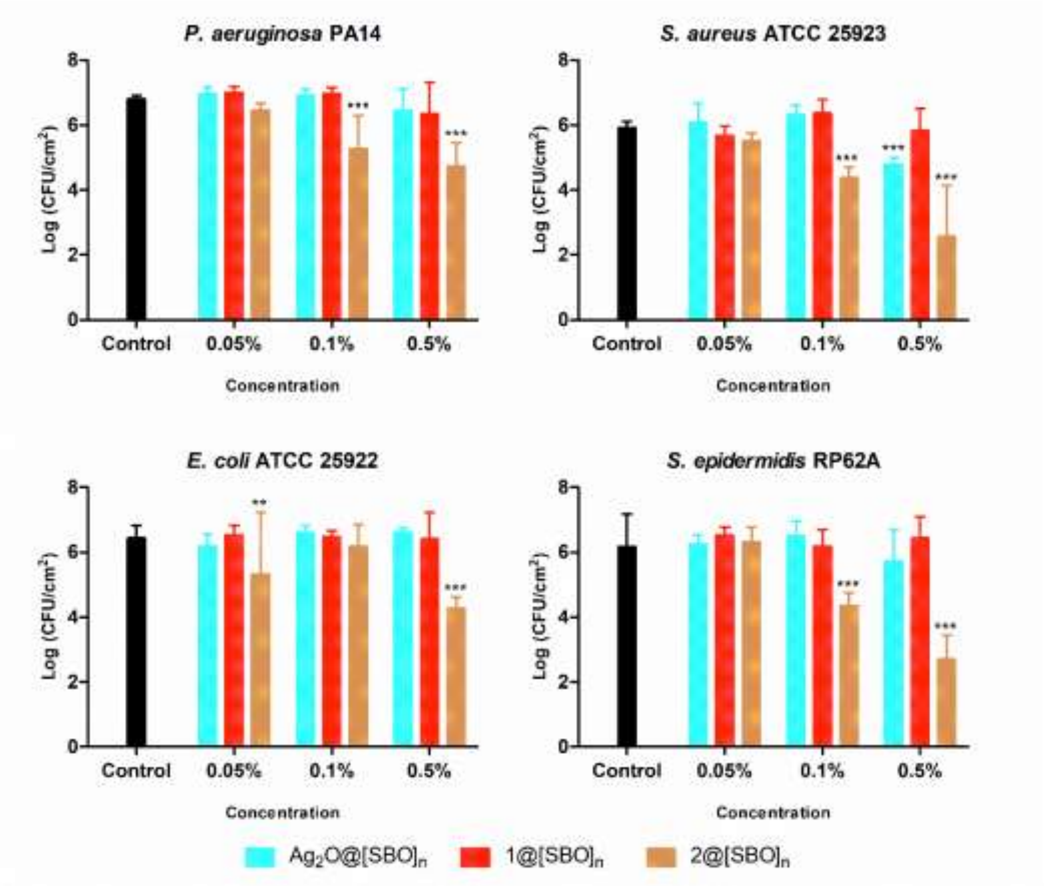

Figure S25. Nonnormalized biofilm growth on $[SBO]_n$ films doped with different concentrations (0.05, 0.1, and 0.5%) of Ag_2O (Ag_2O@$[SBO]_n$) and compounds **1** (**1**@$[SBO]_n$) and **2** (**2**@$[SBO]_n$) against Gram negative (*P. aeruginosa* and *E. coli*) and Gram positive (*S. aureus* and *S. epidermidis*) bacteria. Significant statistical differences were found using the Dunnett's Multiple Comparison Test against the control with 95% confidence interval and are depicted as follows: * - $P \leq 0.05$; ** - $P \leq 0.01$; *** - $P \leq 0.001$.

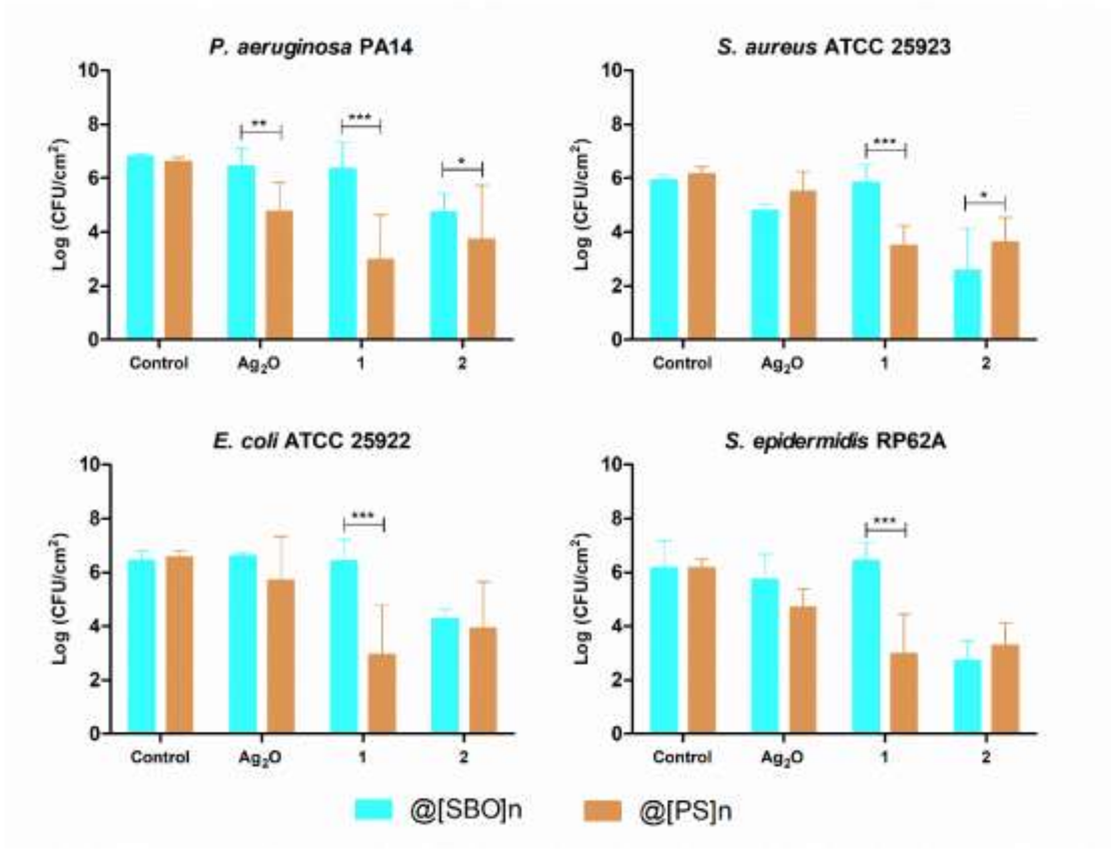

Figure S26. Nonnormalized biofilm inhibition activity of $[SBO]_n$ *vs* $[PS]_n$ films doped with 0.5% of Ag_2O and compounds **1** and **2** against Gram negative (*P. aeruginosa* and *E. coli*) and Gram positive (*S. aureus* and *S. epidermidis*) bacteria. Significant statistical differences were found using the Bonferroni's Multiple Comparison Test with 95% confidence interval and are depicted as follows: * - $P \leq 0.05$; ** - $P \leq 0.01$; *** - $P \leq 0.001$.

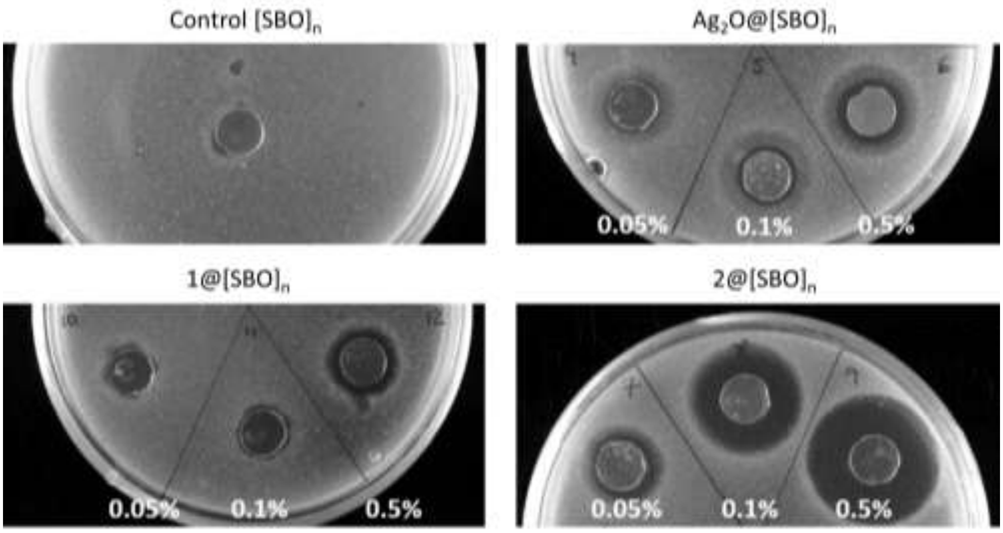

Figure S27. Examples of inhibition halos obtained for *S. epidermidis* RP62A with $[SBO]_n$ films doped with Ag_2O and compounds **1** and **2** (0.05−0.5%).

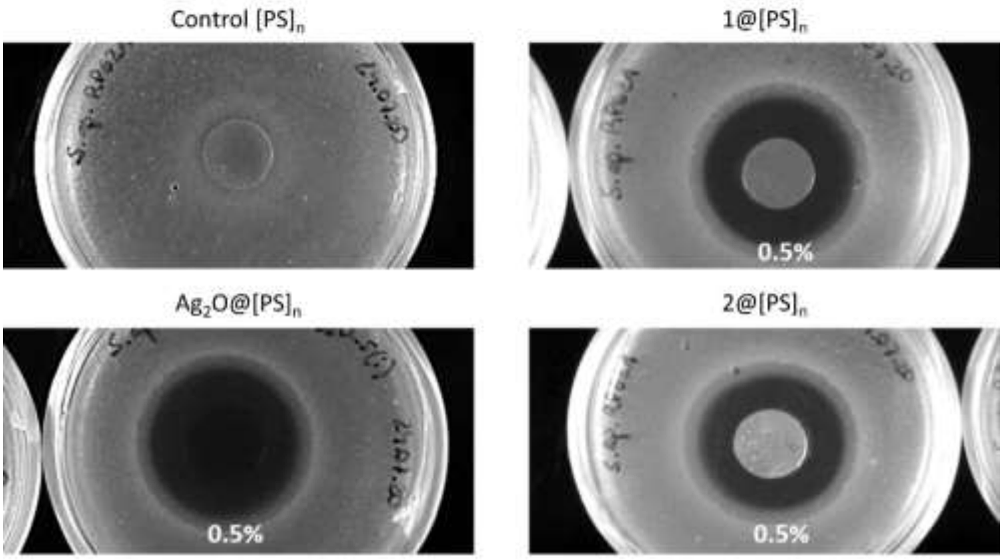

Figure S28. Examples of inhibition halos obtained for *S. epidermidis* RP62A with $[PS]_n$ films doped with Ag_2O and compounds **1** and **2** (0.5%).

Additional References

[1] Macrae, C. F.; Sovago, I.; Cottrell, S. J.; Galek, P. T. A.; McCabe, P.; Pidcock, E.; Platings, M.; Shields, G. P.; Stevens, J. S.; Towler, M.; Wood, P. A. Mercury 4.0: from visualization to analysis, design and prediction. *J. Appl. Cryst.* **2020**, *53*, 226–235.

[2] Sheldrick, G. M. A short history of SHELX. *Acta Crystallogr.* **2008**, *A64*, 112–122.

[3] Farrugia, L. J. WinGX - Version 1.80.05. *J. Appl. Cryst.* **1999**, *32*, 837–838.

[4] Kačuráková, M.; Mathlouthi, M. FTIR and laser-Raman spectra of oligosaccharides in water: characterization of the glycosidic bond. *Carbohydr. Res.* **1996**, *284*, 145–157.

[5] Kizil, R.; Irudayaraj, J.; Seetharaman, K. Characterization of irradiated starches by using FT-Raman and FTIR spectroscopy. *J. Agric. Food Chem.* **2002**, *50*, 3912–3918.

[6] Cael, J. J.; Gardner, K. H.; Koenig, J. L.; Blackwell, J. Infrared and Raman spectroscopy of carbohydrates. Paper V. Normal coordinate analysis of cellulose I. *J. Chem. Phys.* **1975**, *62*, 1145–1153.

[7] Jao, C.-L.; Ko, W.-C. 1,1-Diphenyl-2-picrylhydrazyl (DPPH) radical scavenging by protein hydrolyzates from tuna cooking juice. *Fish. Sci.* **2002**, *68*, 430–435.
